# Incidence, risk factors and outcomes of nosocomial infection in adult patients supported by extracorporeal membrane oxygenation: a systematic review and meta-analysis

**DOI:** 10.1186/s13054-024-04946-8

**Published:** 2024-05-10

**Authors:** Ali Ait Hssain, Amir Vahedian-Azimi, Abdulsalam Saif Ibrahim, Ibrahim Fawzy Hassan, Elie Azoulay, Michael Darmon

**Affiliations:** 1 Medical Intensive Care Unit, Hamad General Hospital, Doha, Qatar; 2 Department of Medicine, Weill Cornell Medical College, Doha, Qatar; 3 College of Health and Life Science, Hamad Bin Khalifa University, Doha, Qatar; 4 Trauma Research Center, Nursing Faculty, Baqiyatallah University of Medical Sciences, Sheykh Bahayi Street, Vanak Square, P.O. Box 19575-174, Tehran, Iran; 5 Médecine Intensive et Réanimation, Hôpital Saint-Louis, Assistance Publique-Hôpitaux de Paris, University of Paris, Paris, France

**Keywords:** Extracorporeal membrane oxygenation, Incidence, Meta-analysis, Nosocomial infection, Prevalence, Risk factors

## Abstract

**Background:**

An increasing number of patients requires extracorporeal membrane oxygenation (ECMO) for life support. This supportive modality is associated with nosocomial infections (NIs). This systematic review and meta-analysis aim to assess the incidence and risk factors of NIs in adult.

**Methods:**

We searched PubMed, Scopus, Web of Science, and ProQuest databases up to 2022. The primary endpoint was incidence of NI. Secondary endpoints included time to infection, source of infection, ECMO duration, Intensive care and hospital length of stay (LOS), ECMO survival and overall survival. Incidence of NI was reported as pooled proportions and 95% confidence intervals (CIs), while dichotomous outcomes were presented as risk ratios (RR) as the effective index and 95% CIs using a random-effects model.

**Results:**

Among the 4,733 adult patients who received ECMO support in the 30 included studies, 1,249 ECMO-related NIs per 1000 ECMO-days was observed. The pooled incidence of NIs across 18 studies involving 3424 patients was 26% (95% CI 14–38%).Ventilator-associated pneumonia (VAP) and bloodstream infections (BSI) were the most common NI sources. Infected patients had lower ECMO survival and overall survival rates compared to non-infected patients, with risk ratio values of 0.84 (95% CI 0.74–0.96, *P* = 0.01) and 0.80 (95% CI 0.71–0.90, *P* < 0.001), respectively.

**Conclusion:**

Results showed that 16% and 20% lower of ECMO survival and overall survival in patients with NI than patients without NI, respectively. However, NI increased the risk of in-hospital mortality by 37% in infected patients compared with non-infected patients. In addition, this study identified the significant positive correlation between ECMO duration and ECMO-related NI.

**Supplementary Information:**

The online version contains supplementary material available at 10.1186/s13054-024-04946-8.

## Background

Extracorporeal membrane oxygenation (ECMO), also known as extracorporeal life support, is an advanced life support modality for critically-ill patients with severe but reversible cardiac and/or respiratory failure [1]. Despite improvements in both technology and management of ECMO, this technique is associated with specific risks and complications [2]. As consequences, many patients treated with ECMO face life-threatening complications such as bleeding, coagulopathy, thrombosis, infection, and stroke [3, 4].

Nosocomial infections (NI) are a common complication in patients treated with ECMO [5, 6]. Main sources of ECMO-related NI include bloodstream infections (BSIs), urinary tract infections (UTIs), surgical site infections (SSIs), and ventilator-associated pneumonia (VAP) [7, 8]. In addition to typical ECMO-related NI, specific ECMO-related infections, such as localized infections at peripheral cannulation insertion sites or mediastinitis in the setting of central cannulation also exists [9–11]. In studies examining different ECMO modalities, including (veno-venous) VV ECMO for respiratory failure and (veno-arterial) VA ECMO for cardiogenic shock, the infection risk was found to range from 8 to 64% [12–15]. Moreover, previous studies have suggested that NIs during ECMO may be related to some predisposing factors, including patients’ underlying condition, the severity of illness, and immunocompromised [16–18]. However, to date, there is no unified understanding of ECMO-related NI from diagnosis to treatment or prevention.

Significant heterogeneity may be expected from existing studies due to differences in case-mix, monocentric design of the performed studies, and inclusion of various ECMO modalities. This systematic review and meta-analysis aim to investigate the incidence of ECMO-related NIs as well as to examine ECMO survival, overall survival and the risk factors related to NI in published studies.

## Methods

### Study design

This systematic review and meta-analysis were performed according to predefined eligibility criteria, search strategies, criteria for study selection and methods for extracting data. It was performed according following the Preferred Reporting Items for Systematic review and Meta-Analysis Protocols (PRISMA-P) 2020 statement [19]. The predefined protocol was registered in the International Prospective Register of Systematic Reviews (PROSPERO) database (CRD42023372412).

### Search strategy and inclusion exclusion criteria

Electronic databases, including PubMed/MEDLINE, Scopus, Web of Science and ProQuest were searched from inception until 1st November 2022. English language publications reporting outcome and clinical characteristics of NI in adult patients receiving ECMO for more than 24 h were selected. To further identify articles for inclusion, all relevant studies and their citations list were examined. The full search strategy is available in Supplementary file 1, Table S1.

The PICOS (Population, Intervention, Comparison, Outcome, and Study type) mnemonic was used for synthesis in this meta-analysis to defined inclusion criteria [20]. Studies were eligible if they met all of the following inclusion criteria: (a) Population: adult (≥ 18 years) patients, male or female; (b) Intervention: supported by ECMO ≥ 24 h; (c) Comparison: compare NI patients with non-NI patients; (d) Outcomes: primary outcome indicators were the prevalence and incidence of NI, and secondary outcome indicators were ECMO survival, survival to hospital discharge, ECMO duration, ICU length of stay (LOS), hospital LOS, microorganism species causing ECMO-related NI, risk factors related to NI and related clinical characteristics of NI and (e) Study type: published retrospective or prospective cohort study. Studies were excluded if (a) studies enrolled patients who had been co-infected before receiving ECMO treatment; (b) studies without access to the full text, publication on animal experiments, review articles, letters-to-the-editor, editorial, case report and conference papers; (c) studies published in non- English languages.

A first screening was performed by title and abstract to identify seemingly related articles. A second screening was performed on selected article after complete assessment of the manuscripts. At each step, assessment was performed independently by two authors (A. AH and A.VA). Disagreement was resolved by discussion and if needed by adjudication by a third author. The final agreement between the three evaluating authors was assessed through Kendall's coefficient of agreement (r = 0.92; *P* < 0.001). Data were extracted from the included studies using a pre-designed form (Supplementary file 2, sheet 1). Moreover, the methodological quality of included manuscripts was assessed [21, 22].

### Quality appraisal

The methodological quality of the included manuscripts was assessed using the JBI critical appraisal tool for cohort studies.. The tool evaluates cohort studies based on 11 criteria, with responses recorded as “Yes”, “No”, “Unclear”, or “Not Applicable”. After evaluating all components of the study, an overall rating was determined based on the number of “Yes” responses: good (≥ 8 yes), medium (5–7 yes), or poor (≤ 4 yes). In addition, the Cochrane Risk of Bias in Observational Studies of Exposures (ROBINS-E) tool was used to evaluate the risk of bias of the included studies [21, 22]. The ROBINS-E tool assesses 7 domains of bias: confounding, selection of participants into the study, classification of exposures, departures from intended exposures, missing data, measurement of outcomes and, selection of the reported result. Domains are classified as low risk of bias, high risk of bias, or unclear risk of bias [23].

### Definition of NI and survival rates

ECMO-related NI was defined according to the Center for Disease Control and Prevention (CDC) as an infection occurring > 24 h after initiation and < 48 h after discontinuation of ECMO [24–26]. Various types of NIs include blood stream infection (BSI), respiratory tract infection (RTI), urinary tract infection (UTI), surgical site infection (SSI), cannula site infection (CSI), and ventilator-associated pneumonia (VAP) [27, 28]. The overall survival rate was defined as the percentage of patients with ECMO who survived to discharge from the hospital out of the total number of patients who received ECMO.

### Primary and secondary outcomes

The primary outcome of this study was the incidence (NI per 1000 ECMO days) of different types of NI in adult patients receiving ECMO. The secondary outcomes included incidence (number of patients developing ≥ 1 episode of NI), time to infection, sources of infection, pathogens, duration of ECMO, ICU and hospital length of stay, ECMO and hospital survival rate.

### Statistical analysis

Descriptive results were reported as percentages, mean ± standard deviation (SD) or median with interquartile range (IQR) calculated from the total number of patients in the analysis. GraphPad Prism 9© (GraphPad Software Inc., La Jolla, CA) and Excel program was used for and forest plots and graphs.

Incidence of NIs and its different types as primary outcomes were reported as pooled proportions and their 95% confidence intervals (CIs), while dichotomous outcomes were presented as pooled risk ratios (RR) and their 95% CIs. In addition, subgroups analysis was carried out based on countries. Due to methodologic variations and sample diversity across studies, the random-effects Linear Mixed Models (REML) was used to extract the pooled estimate. We applied the fixed effect model when the data were homogeneous.

Heterogeneity was assessed using the I-squared (I^2^) statistic, and significance results of the test and values > 50% for I^2^ indicated substantial heterogeneity and the corresponding p-values < 0.05 were also considered as significant [29]. In analyses with significant heterogeneity, a sensitivity analysis and meta-regression analysis were conducted to check the source of heterogeneity. In addition, we used the Galbraith plot to examine heterogeneity [30]. Risk of publication bias was evaluated by visual inspection of funnel plots, the Egger [31] and Begg [32] test were also conducted. Moreover, a nonparametric trim-and-fill method of assessing publication bias was conducted and if there was a publication bias the modified effect size was estimated after adjusting [33]. Finally, we assessed the effect of individual studies on ES, using cumulative analysis based on publication year. Statistical analyses were performed on Review Manager (RevMan) version 5, and STATA version 17 (Stata Corp; College Station; TX, USA). All tests were two-sided y and p-values lower than 0.05 was considered significant.

## Results

### Literature search and manuscript selection

**Fig. 1:**
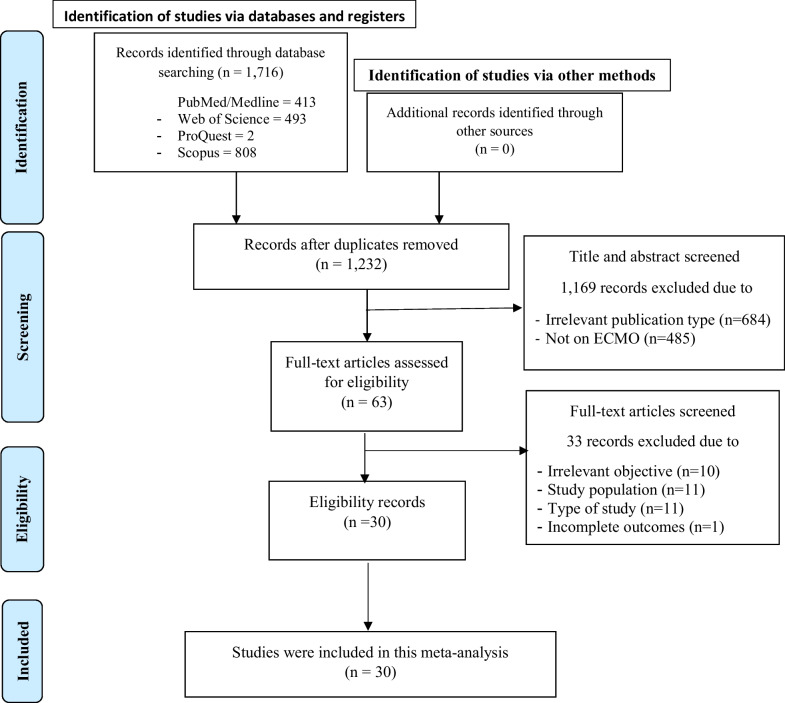
The literature search results and the screening process based on PRISMA 2020 flowchart

The search strategy included PubMed/Medline (*n* = 413), Web of Science (*n* = 493), ProQuest (*n* = 2) and Scopus (*n* = 808) databases resulting in 1,716 studies. After removing duplicates (*n* = 484) and irrelevant studies (*n* = 1169), 63 articles remained for full-text evaluation. Of these, 33 studies were excluded due to an inadequate study population (*n* = 11), inappropriate study design (*n* = 11) or lack of relevant outcome (*n* = 10) (Fig. 1). Details of the 33 excluded studies and the cause for their exclusion are available in Supplementary file 2, sheet 2.

### Quality appraisal results

According to the results of quality assessment, most 24/30 (80%) studies had good quality [6, 8, 10, 12–14, 16, 34–50], and only 6/30 (20%) studies had moderate quality [17, 51–55] (Supplementary file 1, Table S2). In addition, 100% of included studies were classified as having low risk of bias based on Cochrane ROBINS-E tool (Supplementary file 1, Table S3–S4).

### Characteristics of included studies.

Thirty studies with 4733 participants were included. Every one of these studies was of retrospective observational design. Of these participants, 65.4% (3097) were male and the median age was 50 (Range: 18–77). Among the included studies, only one was a multi-center retrospective study [55], while the rest were single-center retrospective studies. Main reason to initiate ECMO was need for cardiac support (63.1%, 2548), respiratory support (33.6%, *n* = 1356), and other causes for the remaining 3.3% (*n* = 135). Most of the studies included both VV ECMO and VA ECMO, four studies focusing only on VA ECMO [12, 16, 42, 51], and three studies on VV ECMO [37, 46, 54] (Tables 1 and 2).

**Table 1:** Demographic and clinical characteristics of studies included according to patients with nosocomial infection (NI) and without NI

Authors names (Refs.)	Publication Year	Research Year	Country	Study population			Age (years)			Male (%)		
				NI group n (%)	Non-NI group n (%)	Total	NI group	non-NI group	*P*-value	NI group	Non-NI group	*P*-value
Hsu et al. [13]	2009	2001–2007	Taiwan	10 (8.8)	104 (91.2)	114	42.1 ± 13.9	52.2 ± 17.3	0.077	50	69.2	0.289
Sun et al. [14]	2010	1996–2007	Taiwan	45 (13.5)	289 (86.5)	334	47 ± 15	52 ± 17	0.099	73.3	67.1	0.407
Schmidt et al. [16]	2012	2003–2009	France	142 (64)	78 (36)	220	49.5 ± 16.9	47.9 ± 15.1	0.460	63	73	0.140
Aubron et al. [8]	2013	2005–2011	Australia	36 (24.6)	110 (75.4)	146	48.5 (37–57)	45 (31–56)	0.453	75	73	0.999
Pieri et al. [34]	2013	2009–2011	Italy	28 (45.9)	33 (54.1)	61	56.9 ± 12.1	57.2 (13.3)	0.900	71.4	75.8	0.700
Kim et al. [35]	2016	2012–2014	Korea	13 (27.7)	34 (72.3)	47	60.2 ± 8.8	50.4 ± 14.9	0.025*	53.8	64.7	0.493
Austin et al. [49]	2017	2011–2014	Australia	21 (21.2)	78 (78.8)	99	48.5 (39–56)	50.5 (40–58)	0.520	57.1	63.3	0.560
Grasselli et al. [17]	2017	2010–2015	Italy	52 (56.5)	40 (43.5)	92	NR	NR	NR	NR	NR	NR
kim et al. [36]	2017	2011–2015	Korea	14 (23)	47 (77)	61	58.5 ± 12.5	61.3 ± 14.9	0.529	50	70.2	0.206
Kutleša et al. [37]	2017	2009–2016	Croatia	35 (35)	65 (65)	100	49 (33–60)	54 (42–64)	0.028*	68.6	66.1	0.806
Sun et al. [38]	2017	2009–2014	China	20 (26.7)	55 (73.3)	75	< 50: 10 (50%)	< 50: 37 (67%)	0.171	55	60	0.697
Bougle et al. [51]	2018	2013–2014	France	85 (55.9)	67 (44.1)	152	> 65: 30 (35%)	> 65: 24 (35%)	> 0.05	81.2	64.2	0.052
Juthani et al. [52]	2018	2012–2015	USA	26 (26)	74 (74)	100	51.2 ± 14.3	52.6 ± 16.2	0.710	57.7	59.4	0.870
Kim et al. [53]	2018	2014–2016	Korea	16 (42.1)	22 (57.9)	38	64.8 ± 9.7	61.5 ± 11.3	0.390	62.5	72.7	0.540
Li et al. [50]	2018	2012–2015	China	30 (40.5)	44 (59.5)	74	51.3 ± 13.1	46.7 ± 18.3	0.983	50	54.5	0.640
Na et al. [54]	2018	2012–2016	Korea	21 (17.4)	100 (82.6)	121	56 (50–65)	60 (51–68)	0.498	57	76	0.078
Allou et al. [10]	2019	2010–2016	France	39 (17.7)	181 (82.3)	220	56 (42–65)	52 (43–63)	0.790	53.8	70.2	0.040*
Menaker et al. [39]	2019	2010–2015	USA	19 (13.1)	126 (86.9)	145	46 (27–61)	44 (29–56)	0.700	57.9	55.6	0.850
Menaker et al. [39]	2019	2010–2015	USA	7 (5.7)	116 (94.3)	123	55 (22–66)	56 (44–64)	0.470	85.7	66.4	0.290
Silvetti et al. [12]	2019	2013–2017	Italy	7 (22.6)	24 (77.4)	31	NR	NR	NR	NR	NR	NR
Ko et al. [40]	2020	2010–2018	Korea	35 (23.3)	115 (76.7)	150	67 (52.5–73)	60 (50–69.5)	0.277	65.7	77.4	0.242
Wang et al. [41]	2020	2013–2019	China	14 (20.3)	55 (79.7)	69	45.5 (18–67)	40 (18–77)	0.403	78.6	58.9	0.222
Wang et al. [42]	2021	2012–2017	China	131 (40.7)	191 (59.3)	322	57.02 ± 12.35	57.3 ± 11.52	0.959	74.81	73.82	0.897
Li et al. [6]	2021	2013–2019	China	16 (28.6)	40 (71.4)	56	51.13 ± 13.18	47.43 ± 15.1	0.400	50	62.5	0.390
Quintana et al. [43]	2021	2015–2017	USA	37 (18)	169 (82)	206	52 ± 16	54 ± 15	0.418	19	81	0.610
Quintana et al. [43]	2021	2015–2017	USA	18 (24)	57 (76)	75	50 ± 19	48 ± 18	0.596	26.2	73.8	0.616
Selçuk et al. [44]	2021	2012–2016	Turkey	27 (45)	33 (55)	60	53.3 ± 18.8	54.5 ± 15.8	0.895	NR	NR	NR
Lee et al. [45]	2022	2015–2021	Korea	97 (20.8)	368 (79.2)	465	58.9 ± 13.3	55.5 ± 14.9	0.031*	64.9	65.8	0.881
Lee et al. [45]	2022	2015–2021	Korea	26 (6.4)	368 (93.4)	394	58.5 ± 12.7	55.5 ± 14.9	0.323	53.8	65.8	0.215
Solla-Buceta et al. [55]	2022	2010–2015	Spain	87 (34.9)	162 (65.1)	249	51.5 ± 12.3	49.5 ± 12.5	0.239	79.3	74.7	0.414
Manerikar et al. [46]	2022	2016–2019	USA	15 (24.6)	46 (75.4)	61	45.5 ± 14.7	48.1 ± 15.7	0.560	NR	NR	NR
Xu et al. [47]	2022	2011–2020	China	42 (53.2)	37 (46.8)	79	57.3 ± 13.9	48.8 ± 15.5	0.014*	67	65	0.870
Zang et al. [48]	2022	2013–2020	China	38 (19.6)	156 (80.4)	194	46.9 ± 16.7	47.6 ± 17.9	0.935	57.89	62.18	0.627

**Table 2:** Type of supported device, cause of ECMO, ECMO mode in participants of studies included

Authors names (Refs.)	Type of supported device and time (hours)	Cause of ECMO			Veno-arterial (VA) ECMO			Veno-venous (VV) ECMO			*P*-value
		Respiratory (%)	Cardiogenic (%)	Others (%)	NI group (%)	non-NI group (%)	Total (%)	NI group (%)	non-NI group (%)	Total (%)	
Hsu et al. [13]	ECMO > 72	19.3	80.7	0	80	90	83.25	20	12.5	16.25	> 0.05
Sun et al. [14]	ECMO > 48	20.4	79.6	0	60	83.5	71.75	37.8	14	25.9	0.001
Schmidt et al. [16]	ECMO and MV > 48	3.6	96.4	0	100	100	100	0	0	0	0
Aubron et al. [8]	ECMO and MV > 48	34.2	65.8	0	75	63	138	25	37	31	0.226
Pieri et al. [34]	ECMO and MV > 48	36.1	62.3	1.6	39.3	39.4	69	25	45.5	35.25	0.1
Kim et al. [35]	ECMO > 48	NR	NR	NR	31.8	68.2	50	24	76	50	> 0.05
Austin et al. [49]	ECMO > 48	17.2	82.8	0	85.7	44.9	65.3	4.8	41	22.9	0.001*
Grasselli et al. [17]	ECMO and MV > 24	82.6	6.5	10.9	NR	NR	20	NR	NR	80	NR
kim et al. [36]	VA ECMO > 48	NR	NR	NR	NR	NR	95.1	NR	NR	4.9	NR
Kutleša et al. [37]	VA ECMO > 48	72.0	0	28	0	0	0	100	100	100	0
Sun et al. [38]	ECMO > 24	NR	NR	NR	85	94.5	89.7	5.5	15	10.25	0.386
Bougle et al. [51]	VA ECMO and MV > 48	94.1	5.9	0	100	100	100	0	0	0	0
Juthani et al. [52]	ECMO > 48	48.0	36	16	34.6	36.5	35.5	65.4	63.5	64.4	0.86
Kim et al. [53]	ECMO > 48	0	100	0	NR	NR	92.1	NR	NR	7.9	NR
Li et al. [50]	ECMO > 48	0	100	0	90	86.4	88.2	10	11.4	10.7	0.827
Na et al. [54]	VV ECMO > 48	71.9	0	28.1	0	0	0	100	100	100	0
Allou et al. [10]	ECMO > 48	28.6	71.4	0	76.9	66.9	71.9	23.1	33.1	28.1	0.210
Menaker et al. [39]	VV ECMO > 48	NR	NR	NR	0	0	0	100	100	100	0
Menaker et al. [39]	VA ECMO > 48	43.1	56.9	0	100	100	100	0	0	0	0
Silvetti et al. [12]	VA ECMO > 48	0	100	0	100	100	100	0	0	0	0
Ko et al. [40]	VA ECMO > 24	0	100	0	100	100	100	0	0	0	0
Wang et al. [41]	ECMO > 48	17.4	75.4	7.2	50	92.7	71.9	50	7.3	28.6	0.001*
Wang et al. [42]	VA ECMO > 48	0	100	0	100	100	100	0	0	0	0
Li et al. [6]	ECMO > 48	NR	NR	NR	93.75	92.5	93.1	6.25	7.5	6.9	0.870
Quintana et al. [43]	VA ECMO > 48	26.2	70.4	3.4	100	100	100	0	0	0	0
Quintana et al. [43]	VV ECMO > 48	38.7	16	45.3	0	0	0	100	100	100	0
Selçuk et al. [44]	ECMO > 48	3.3	96.7	0	NR	NR	96.8	NR	NR	3.2	NR
Lee et al. [45]	ECMO > 48	44.1	55.9	0	37.1	54.9	46	26.8	26.4	26.6	0.993
Lee et al. [45]	ECMO > 48	42.9	57.1	0	30.8	54.9	42.8	38.5	26.4	32.4	0.160
Solla-Buceta et al. [55]	ECMO > 48	NR	NR	NR	46.08	68.01	57.01	54.02	31.99	43.01	0.001*
Manerikar et al. [46]	VV ECMO > 48	NR	NR	NR	0	0	0	100	100	100	0
Xu et al. [47]	ECMO > 48	65.8	34.2	0	10	38	24	90	62	76	0.003*
Zang et al. [48]	ECMO > 48	53.6	46.4	0	NR	NR	NR	NR	NR	NR	NR

### Descriptive results

Clinical outcomes available in included studies are reported in Supplementary file 1, Table S5–S9. A comprehensive analysis of 30 studies involving 4733 adult patients on ECMO treatment revealed that there were 1249 ECMO-related NIs per 1000 ECMO-days. Males accounted for 60.93% of infected patients, with an average age of 53.17 ± 13.95 years. Hypertension was the most common underlying condition in both infected and non-infected patients (Supplementary file 1, Figure S1). Patients with NIs had significantly longer ECMO, ICU, and hospital stays (Supplementary file 1, Figure S1). The total incidence range of NIs was 4.1–85.4% with 2059 pathogens identified from 1,498 NI episodes in 1249 infected patients. The incidence of ECMO-related NI was 2.98–24.7% for BSI, 3.97–17% for SSI, 3.97–24.7% for RTI, 1.99–31% for UTI, 23.9–55.4% for VAP, and 7.1–11% for CSI. Gram-negative bacteria were identified as the most prevalent pathogens (48.6%), followed by Gram-positive bacteria and fungi. *Acinetobacter baumannii*, *Pseudomonas aeruginosa*, and *Klebsiella pneumoniae* were the most common Gram-negative bacteria, while *Enterococcus spp*., *Coagulase-negative Staphylococcus*, and *Staphylococcus aureus* were the predominant Gram-positive bacteria.

### Risk factors for NI

Results showed that the MV duration, hospital LOS, ECMO mode (VV ECMO vs. VA ECMO), having underlying diseases (yes vs. no), mechanical complication, SOFA score, SAPS score, ECMO catheter colonization, age, duration of arterial catheter, acute renal failure, acute hepatic failure, body mass index (MBI), cardiopulmonary resuscitation (CPR) < 5 min and hemodialysis were significantly increased the risk of NI (Supplementary file 1, Figure S2B). According to pooled analysis in the current study, the cumulative odds ratio of ECMO duration to predict NI was 1.05 (95%CI 1.02–1.08, *P* < 0.001), with substantial significant heterogeneity between studies (I^2^ = 98.8%, *P* < 0.001) (Supplementary file 1, Figure S3).

### Primary outcomes

**Fig. 2:**
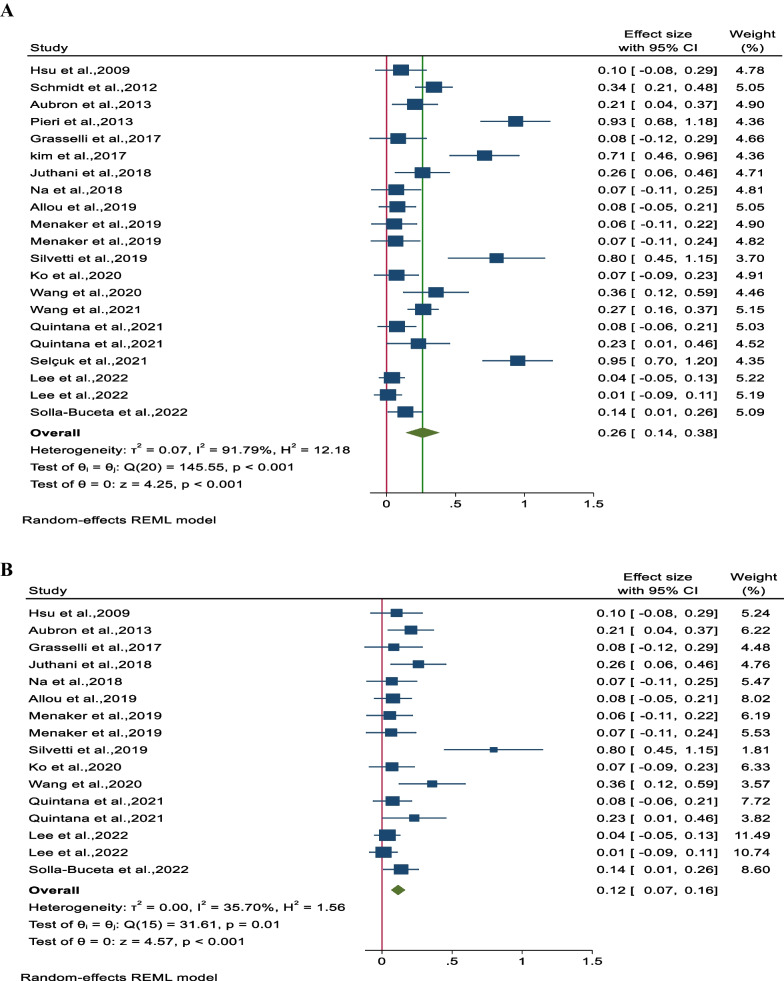
Forest plot for **A** pooled incidence NIs per 1000 ECMO-day and **B** pooled incidence of NIs per 1000 ECMO-day after reducing heterogeneity

The pooled incidence rate of NIs, as reported in 18 studies involving 3,424 patients, was found to be 0.26 (95% CI 0.14–0.38, *P* < 0.001), indicating a statistically significant result. However, there was substantial heterogeneity observed between the studies (I^2^ = 91.8%, *P* < 0.001) (Fig. 2A). To address this heterogeneity, a sensitivity analysis was conducted where the study or studies causing the heterogeneity were excluded. Upon recalculating, the adjusted pooled incidence of NI (based on 13 studies and 2,761 patients) was determined to be 0.12 (95% CI 0.07–0.16, *P* < 0.001) with a mild heterogeneity (I^2^ = 35.7%, *P* = 0.01) (Fig. 2B).

### Secondary outcomes

#### ECMO survival

**Fig. 3:**
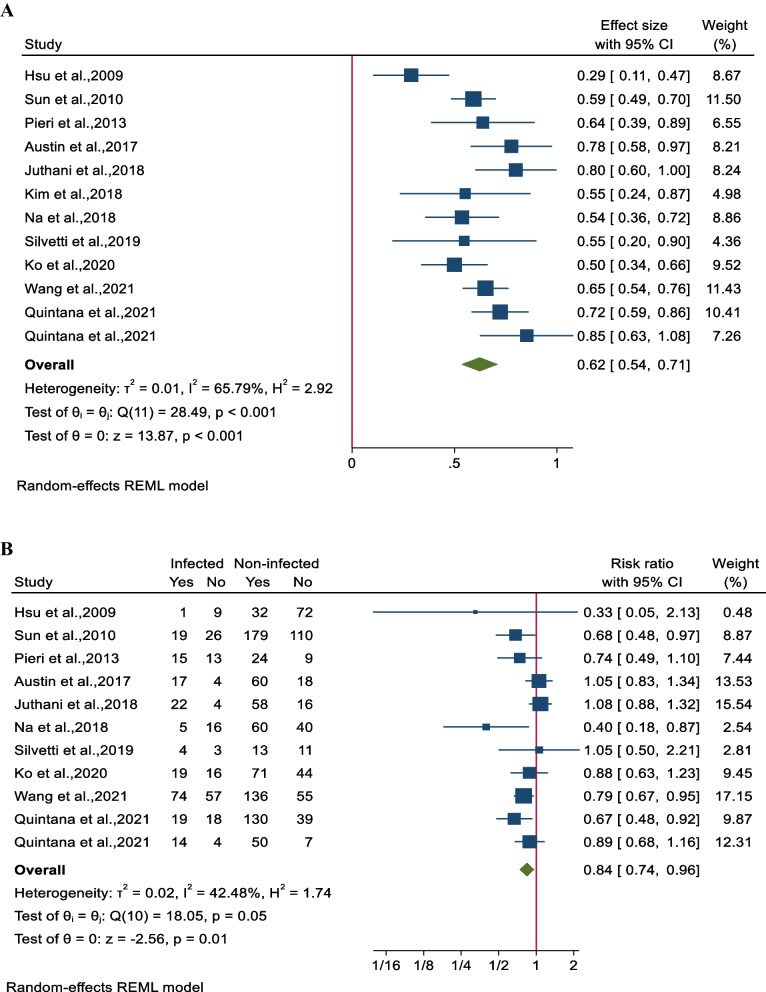
Forest plot for pooled ECMO survival rates for **A** all participants in each study and **B** between infected and non-infected patients

The survival rate of patients undergoing combined ECMO treatment was determined to be 62% (95% CI 54–70; 11 studies involving 1651 participants). Notably, there was substantial heterogeneity among the studies (I^2^ = 65.8%, *P* < 0.001 (Fig. 3A). The impact of nosocomial infections on ECMO survival was assessed in 10 studies involving 1613 patients. It was found that ECMO survival rates were significantly lower in patients with NIs, with a pooled risk ratio (RR) of 84% (95% CI 74–96%). A moderate level of heterogeneity was observed among the studies (I^2^ = 42.5%, *P* = 0.05) (Fig. 3B).

### Overall survival

**Fig. 4:**
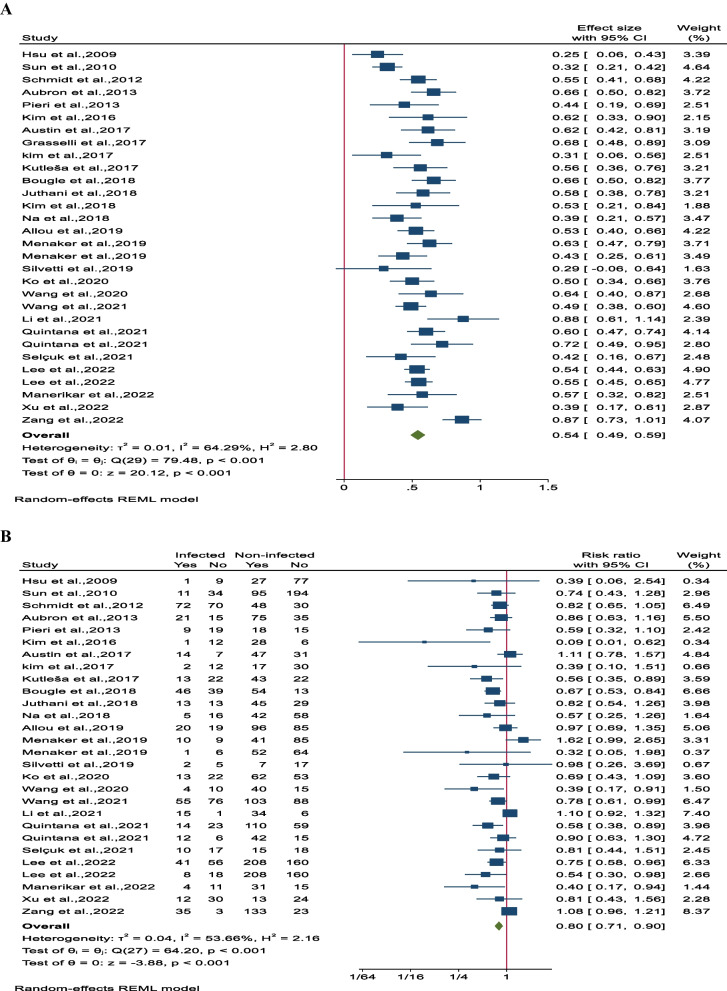
Forest plot of overall survival rates for **A** all participants in each study and **B** between infected and non-infected patient

The overall survival rate was determined to be 54% (95% CI 49–59; 11 studies involving 1651 participants). Notably, there was significant heterogeneity observed among the studies (I^2^ = 64.5%, *P* < 0.001; Fig. 4A). Comparing the overall survival rates between the nosocomial infection (NI) group and control patients revealed a substantial difference, with the NI group showing a lower survival rate of 80% (95% CI 71–90; 24 studies involving 4205 patients). There was also notable heterogeneity among the studies (I^2^ = 53.7%, *P* < 0.001, Fig. 4B). Additionally, detailed subgroup analysis, sensitivity analysis, and assessment of publication bias can be found in Supplementary File 3, Figs. 1A–5D.

### Time trend

Influence of NI on outcome was not affected by publication date (Supplementary file 1, Figure S4A). NI rate was however associated with the publication date (Supplementary file 1, Figure S4B). However, overall survival was lower among studies published from 2009 to 2013 (Supplementary file 1, Figure S5A–S5B).

### Meta-regression

In order to explore the heterogeneity, a meta-regression analysis was conducted. Mortality was found to be linked to factors such as patients' severity of illness based on APACHE II scores, age, and VV ECMO, while ECMO survival was associated with nosocomial infections. The findings of the meta-regression analysis are presented in Supplementary File 1, Table S10. Additionally, a forest plot depicting the impact of these variables on the outcomes is included in Supplementary File 4, Figures S1–S19.

## Discussion

This systematic review and meta-analysis aimed to assess the overall incidence of ECMO-related NIs and their impact on mortality, offering a comprehensive evaluation. Across 18 studies involving 3,424 patients, the pooled incidence of NIs was 26%. The time to the first NI ranged from 3 to 15.6 days after ECMO initiation, with a notable number of patients developing NIs beyond two weeks [16, 17, 44]. The incidence of NIs varied widely among studies, ranging from 4.1% to 85.4%. This variability could be attributed to factors like case mix, diagnostic criteria, reporting systems, antibiotic prophylaxis strategies, and center-specific effects [5, 56, 57].

The incidence of ECMO-related NIs and their impact on outcomes in patients supported by ECMO have been previously reported in literature. Studies have shown that the rate of infection can vary, with reports ranging from 8 to 46%. Previous reviews of the Extracorporeal Life Support Organization (ELSO) registry by Bizzarro et al. [58], and Vogel et al. [59], found rates of infection to be 11.7% and 10.2%, respectively, which is lower than the rates seen in our study. This discrepancy among studies may be attributed to differences in study populations, methodologies, variations in infection prevention practices, the emergence of new pathogens and antimicrobial resistance, as well as improvements in surveillance and reporting methods [60, 61].

Overall, 2059 pathogens were isolated from 1498 NI episodes in 1249 (26.4%) infected patients. Our findings identified VAP (33%), BSI (15%), and RTI (15%) as the most common ECMO-related NIs, primarily caused by GNB like *Acinetobacter baumannii, Pseudomonas aeruginosa,* and *Klebsiella pneumoniae*. Studies indicate VAP rates ranging from 10.7 to 54.5%, mainly attributed to GNB such as *Pseudomonas aeruginosa*, *Klebsiella pneumoniae*, and *Acinetobacter species*, and GPB like *Staphylococcus aureus* [8, 16, 17, 40, 47, 51]. BSI prevalence in adult ECMO patients ranges from 2.6 to 44.7%, with GBP, especially *coagulase-negative staphylococci* and *Staphylococcus aureus*, being the primary pathogens, followed by GNB (10–20%) such as *Acinetobacter baumannii* and *Pseudomonas aeruginosa*, and fungal infections like *Candida spp.* [13, 37, 39]. RTI rates vary from 1.1 to 32.1%, primarily caused by GNB like *Klebsiella pneumoniae*, *Pseudomonas aeruginosa*, and *Haemophilus influenza* [16, 51].

The study, consistent with the previous work by Li et al. [5], identified several risk factors for NIs in adult patients undergoing ECMO. These risk factors included the duration of MV, length of hospital stay, ECMO mode, underlying diseases, disease severity, ECMO catheter colonization, patient age, duration of arterial catheter placement, acute renal failure, acute hepatic failure, BMI, ECPR exceeding 5 min, hemodialysis, and mechanical complications. Patients supported by VV ECMO exhibited a higher susceptibility to developing Nosocomial Infections (NIs) compared to those on VA ECMO [14, 41, 47]. Despite this, the VA ECMO modality is recognized for its increased complexity, entailing higher risks of vascular trauma, systemic embolization, and ischemia [62]. The exact reason behind the heightened NI risk in VV ECMO patients remains somewhat ambiguous. This elevated risk may be linked to the prolonged ECMO treatment and duration of ventilator support in VV ECMO patients [14, 41]. Additionally, the longer duration of VV ECMO in lung transplant recipients inherently exposes them to an extended period of susceptibility to NIs, potentially leading to skewed infection rates and outcomes when contrasted with heart transplant patients supported by VA ECMO with shorter durations of support. The study highlights a significant association between NIs and adverse outcomes in adult ECMO patients, resulting in a relative risk reduction of 16% in ECMO survival rates and 20% in overall survival rates. Moreover, NIs were found to elevate the relative risk of hospital mortality, particularly in cases of prolonged ECMO duration, which showed a potential four-fold increase in NI risk [34, 37, 38, 42, 45, 47, 52, 55].This heightened risk can be attributed to the critical condition of patients on long-term ECMO, prolonged exposure to risks, and the intensity of invasive care. Time-dependent bias is a critical consideration in studies involving ECMO duration and nosocomial infections. In the context of ECMO, the duration of ECMO support can act both as a risk factor for developing infections and as a consequence of infection occurrence. Prolonged ECMO duration has been associated with an increased risk of nosocomial infections due to factors such as prolonged exposure to invasive devices, prolonged hospitalization, and compromised immune function [63]. Longer ECMO duration not only increases the likelihood of acquiring infections but can also be a consequence of infections that prolong the need for ECMO support. This bidirectional relationship underscores the complexity of managing infections in ECMO patients and emphasizes the need for vigilant monitoring, infection prevention strategies, and timely interventions to mitigate the risks associated with prolonged ECMO support.

The observed increase in NI rates in more recent studies [45–48], despite older studies showing lower survival rates is indeed a noteworthy finding [13, 14, 16]. This apparent discrepancy does not necessarily negate the conclusion that NIs can impact mortality in ECMO patients. Instead, it may reflect improved surveillance, detection, and reporting of NIs over time. One plausible explanation for this occurrence could be advancements in critical care practices and infection control measures over time. With improvements in healthcare protocols, including enhanced sepsis management, antimicrobial stewardship, and ECMO circuit technology, it is possible that while NI rates have risen in recent years, overall survival rates have improved due to better management of infections. Moreover, the evolving landscape of ECMO therapy, including patient selection criteria, cannulation techniques, and anticoagulation strategies, may have influenced both NI rates and patient outcomes over time. The study could be useful for clinicians and researchers regarding infection risk factors in ECMO patients. Further studies aiming at identifying high-risk patients are needed so that clinicians and researchers can pinpoint high-risk patients for tailored monitoring and interventions.

This study has several limitations that should be considered. Firstly, the retrospective and single-center nature of most included studies, along with small sample sizes, limits data availability on confounding factors and the establishment of appropriate exposure and comparison groups. Secondly, there was significant heterogeneity due to variations in case mix, nosocomial infection rates, and management practices across different centers. Thirdly, a notable limitation is the inadequate consideration of time dependency of nosocomial infections in most studies, potentially leading to misleading associations between ECMO/ICU duration and infection outcomes. Lastly, the potential impact of changes in sepsis definitions and management practices over the years on the identification of BSI in ECMO patients is a critical consideration. These evolving standards may introduce variability in how infections are identified and managed, which could affect the study's outcomes. To address these limitations, we have conducted sensitivity analyses to mitigate potential biases arising from these changes, ensuring the robustness of our results. These limitations highlight the need for future research to address these gaps and improve our understanding of the impact of NIs on patient outcomes.

## Conclusion

This study highlights a heightened risk of NIs, particularly Ventilator-VAP, BSI, and RTI, in patients undergoing ECMO for refractory respiratory or cardiogenic failure. The pooled analysis revealed a 26% incidence rate per 1000 ECMO-days of NIs in adult ECMO patients. Our findings indicate a 16% and 20% lower ECMO survival and overall survival, respectively, in patients with NIs compared to those without. The dynamic nature of ECMO therapy, encompassing evolving patient selection criteria, cannulation techniques, and anticoagulation strategies, may have impacted both NI rates and patient outcomes. Further research is warranted to delve deeper into assessing the risk of nosocomial infections while considering time-dependent confounders, evaluating the efficacy of prevention strategies, and understanding their impact on both infection rates and outcomes.

## Supplementary Information

Additional file 1 (DOCX 793 KB)

Additional file 2 (XLSX 72 KB)

Additional file 3 (DOCX 217 KB)

Additional file 4 (DOCX 2245 KB)

## Data Availability

The data that support the findings of this study are available from the corresponding author upon reasonable request.

## Abbreviations

ECMO: Extracorporeal membrane oxygenation
NIs: Nosocomial infections
BSIs: Bloodstream infections
UTIs: Urinary tract infections
SSIs: Surgical site infections
VAP: Ventilator-associated pneumonia
CSI: Cannula site infection
VA ECMO: Venoarterial extracorporeal membrane oxygenation
VV ECMO: Venovenous extracorporeal membrane oxygenation
LOS: Length of stay
CIs: Confidence intervals
RR: Risk ratios
REML: Linear Mixed Models
