# Supplementary material for: Incidence, risk factors and outcomes of nosocomial infection in adult patients supported by extracorporeal membrane oxygenation: a systematic review and meta-analysis
Source: Crit Care. 2024 May 10;28:158. doi: 10.1186/s13054-024-04946-8 (PMC11088079; doi:10.1186/s13054-024-04946-8)
Supplement: Supplementary file 1 — Additional file 1 (DOCX 793 KB) [file 13054_2024_4946_MOESM1_ESM.docx]

**Table 1S:** Search of study

| **Scopus: 808** ( TITLE-ABS-KEY ( "Extracorporeal Membrane Oxygenation" OR "oxygenators, membrane" OR "Extracorporeal Membrane Oxygenation" OR "ECMO" OR "extracorporeal life support" OR "ECLS" OR "membrane oxygenator*" ) AND TITLE-ABS-KEY ( "Bacteraemia" OR "Catheter-Related Infections" OR "Cross Infection" OR "Fungemia" OR "Gram-Positive Bacterial Infections" OR "pneumonia, ventilator associated" OR "Shock, Septic" OR "Urinary Tract Infections" OR "Candidemia" OR "bloodstream infection*" OR "catheter related infection*" OR "catheter associated infection*" OR "device associated infection*" OR "fungal infection*" OR "cross infection*" OR "healthcare associated infection*" OR "hospital infection*" OR "nosocomial infection*" OR "fungemia*" OR "pseudomembranous colitis" OR "ventilator-associated pneumonia" OR "urinary tract infection*" OR "septic shock" ) AND TITLE-ABS-KEY ( "Duration of Therapy" OR "Length of stay" OR "Mortality" OR "Survival Rate" OR "Duration of Therapy" OR "therapy duration" OR "duration of treatment" OR "treatment duration" OR "death rate*" OR "Length of stay" OR "stay length" OR "mortality*" OR "survival" OR "incidence")  **PubMed/Medline: 413** (("Extracorporeal Membrane Oxygenation"[Title/Abstract] OR "oxygenators, membrane"[Title/Abstract] OR "ECMO"[Title/Abstract] OR "extracorporeal life support"[Title/Abstract] OR "ECLS"[Title/Abstract] OR "membrane oxygenator*"[Title/Abstract] ) AND ("Bacteraemia"[Title/Abstract] OR "Catheter-Related Infections"[Title/Abstract] OR "Cross Infection"[Title/Abstract] OR "Fungemia"[Title/Abstract] OR "Gram-Positive Bacterial Infections"[Title/Abstract] OR "pneumonia, ventilator associated"[Title/Abstract] OR "Shock, Septic"[Title/Abstract] OR "Urinary Tract Infections"[Title/Abstract] OR "Candidemia"[Title/Abstract] OR "bloodstream infection*"[Title/Abstract] OR "catheter related infection*"[Title/Abstract] OR "catheter associated infection*"[Title/Abstract] OR "device associated infection*"[Title/Abstract] OR "fungal infection*"[Title/Abstract] OR "cross infection*"[Title/Abstract] OR "healthcare associated infection*"[Title/Abstract] OR "hospital infection*"[Title/Abstract] OR "nosocomial infection*"[Title/Abstract] OR "fungemia*"[Title/Abstract] OR "pseudomembranous colitis"[Title/Abstract] OR "ventilator-associated pneumonia"[Title/Abstract] OR "urinary tract infection*"[Title/Abstract] OR "septic shock"[Title/Abstract])) AND ("Duration of Therapy"[Title/Abstract] OR "Length of stay"[Title/Abstract] OR "Mortality"[Title/Abstract] OR "Survival Rate"[Title/Abstract] OR "therapy duration"[Title/Abstract] OR "duration of treatment"[Title/Abstract] OR "treatment duration"[Title/Abstract] OR "death rate*"[Title/Abstract] OR "stay length"[Title/Abstract] OR "mortality*"[Title/Abstract] OR "survival"[Title/Abstract] OR "incidence"[Title/Abstract] OR OR[Title/Abstract])  **Web of Science: 493** https://www.webofscience.com/wos/woscc/summary/ce22c216-167b-4a5b-b3f7-9353d7588bf1-5128b538/relevance/1  **ProQuest: 2** ("Extracorporeal Membrane Oxygenation" OR "oxygenators, membrane" OR "Extracorporeal Membrane Oxygenation" OR "ECMO" OR "extracorporeal life support" OR "ECLS" OR "membrane oxygenator*" ) AND ("Bacteraemia" OR "Catheter-Related Infections" OR "Cross Infection" OR "Fungemia" OR "Gram-Positive Bacterial Infections" OR "pneumonia, ventilator associated" OR "Shock, Septic" OR "Urinary Tract Infections" OR "Candidemia" OR "bloodstream infection*" OR "catheter related infection*" OR "catheter associated infection*" OR "device associated infection*" OR "fungal infection*" OR "cross infection*" OR "healthcare associated infection*" OR "hospital infection*" OR "nosocomial infection*" OR "fungemia*" OR "pseudomembranous colitis" OR "ventilator-associated pneumonia" OR "urinary tract infection*" OR "septic shock" ) AND ("Duration of Therapy" OR "Length of stay" OR "Mortality" OR "Survival Rate" OR "Duration of Therapy" OR "therapy duration" OR "duration of treatment" OR "treatment duration" OR "death rate*" OR "Length of stay" OR "stay length" OR "mortality*" OR "survival" OR "incidence" |
| --- |

**Table S2:** Joanna Briggs Institute (JBI) checklists for cohort studies to quality assessment of included studies

| Study  (1^st^ author, year, Ref) | Question no. | | | | | | | | | | | Overall score |
| --- | --- | --- | --- | --- | --- | --- | --- | --- | --- | --- | --- | --- |
|  | **1** | **2** | **3** | **4** | **5** | **6** | **7** | **8** | **9** | **10** | **11** |  |
| Hsu et al. 2009, [1] | Y | Y | Y | Y | N | Y | Y | Y | Y | NA | Y | 9 (Good) |
| Sun et al. 2010, [2] | Y | Y | Y | Y | N | Y | Y | Y | Y | NA | Y | 9 (Good) |
| Schmidt et al. 2012, [3] | Y | Y | Y | Y | Y | Y | Y | Y | Y | NA | Y | 10 (Good) |
| Aubron et al. 2013, [4] | Y | Y | Y | Y | N | N | Y | Y | Y | NA | Y | 8 (Good) |
| Pieri et al. 2013, [5] | Y | Y | Y | Y | N | Y | Y | Y | Y | NA | Y | 9 (Good) |
| Kim et al. 2016, [6] | Y | Y | Y | Y | N | Y | Y | Y | Y | NA | Y | 9 (Good) |
| Austin et al. 2017, [7] | Y | Y | Y | N | N | Y | Y | Y | Y | NA | Y | 8 (Good) |
| Grasselli et al. 2017, [8] | Y | Y | Y | N | N | Y | Y | Y | N | NA | Y | 7 (Medium) |
| kim et al. 2017, [9] | Y | Y | Y | Y | N | Y | Y | Y | Y | NA | Y | 9 (Good) |
| Kutleša et al. 2017, [10] | Y | Y | Y | N | N | Y | Y | Y | Y | NA | Y | 8 (Good) |
| Sun et al. 2017, [11] | Y | Y | Y | Y | N | Y | Y | Y | N | NA | Y | 8 (Good) |
| Bougle et al. 2018, [12] | Y | Y | Y | N | N | Y | Y | Y | N | NA | Y | 7 (Medium) |
| Juthani et al. 2018, [13] | Y | Y | Y | N | N | Y | Y | Y | N | NA | Y | 7 (Medium) |
| Kim et al. 2018, [14] | Y | Y | Y | N | N | N | Y | Y | N | NA | Y | 6 (Medium) |
| Li et al. 2018, [15] | Y | Y | Y | Y | N | Y | Y | Y | N | NA | Y | 8 (Good) |
| Na et al. 2018, [16] | Y | Y | Y | N | N | Y | Y | Y | N | NA | Y | 7 (Medium) |
| Allou et al. 2019, [17] | Y | Y | Y | Y | N | Y | Y | Y | Y | NA | Y | 9 (Good) |
| Menaker et al. 2019, [18] | Y | Y | Y | N | N | Y | Y | Y | Y | NA | Y | 8 (Good) |
| Silvetti et al. 2019, [19] | Y | Y | Y | N | Y | Y | Y | Y | Y | NA | Y | 9 (Good) |
| Ko et al. 2019, [20] | Y | Y | Y | Y | Y | Y | Y | Y | Y | NA | Y | 10 (Good) |
| Wang et al. 2020, [21] | Y | Y | Y | N | Y | Y | Y | Y | Y | NA | Y | 9 (Good) |
| Wang et al. 2021, [22] | Y | Y | Y | Y | Y | Y | Y | Y | Y | NA | Y | 10 (Good) |
| Li et al. 2021, [23] | U | Y | Y | N | Y | Y | Y | Y | Y | NA | Y | 8 (Good) |
| Quintana et al. 2021, [24] | Y | Y | Y | N | N | Y | Y | Y | Y | NA | Y | 8 (Good) |
| Selçuk et al. 2021, [25] | Y | Y | Y | N | N | Y | Y | Y | Y | NA | Y | 8 (Good) |
| Lee et al. 2022, [26] | Y | Y | Y | Y | Y | Y | Y | Y | Y | NA | Y | 10 (Good) |
| Solla-Buceta et al. 2022, [27] | Y | Y | Y | N | Y | Y | Y | N | N | NA | Y | 7 (Medium) |
| Manerikar et al. 2022, [28] | Y | Y | Y | N | Y | Y | Y | Y | Y | NA | Y | 9 (Good) |
| Xu et al. 2022, [29] | Y | Y | Y | Y | Y | Y | Y | Y | Y | NA | Y | 10 (Good) |
| Zang et al. 2022, [30] | Y | Y | Y | Y | Y | Y | Y | Y | Y | NA | Y | 10 (Good) |

Answers: Yes, No, Unclear or Not/Applicable; Based on the number of "Yes" responses, a rating of good = (≥8 yes), medium= (5-7) and poor = (≤4 yes). Questions; 1. Were the two groups similar and recruited from the same population? 2. Were the exposures measured similarly to assign people to both exposed and unexposed groups? 3. Was the exposure measured in a valid and reliable way? 4. Dose confounding factors identified? 5. Were strategies to deal with confounding factors stated? 6. Were the groups/participants free of the outcome at the start of the study (or at the moment exposure)? 7. Were the outcomes measured in a valid and reliable way? 8. Was the follow up time reported and sufficient to be long enough for outcomes to occur? 9. Was follow up complete, and if not, were the reasons to loss to follow up described and explored? 10. Were strategies to address incomplete follow up utilized? 11. Was appropriate statistical analysis used?

**Table S3:** Risk of bias of included studies based on Cochrane Risk of Bias in Observational Studies of Exposures (ROBINS-E) tool


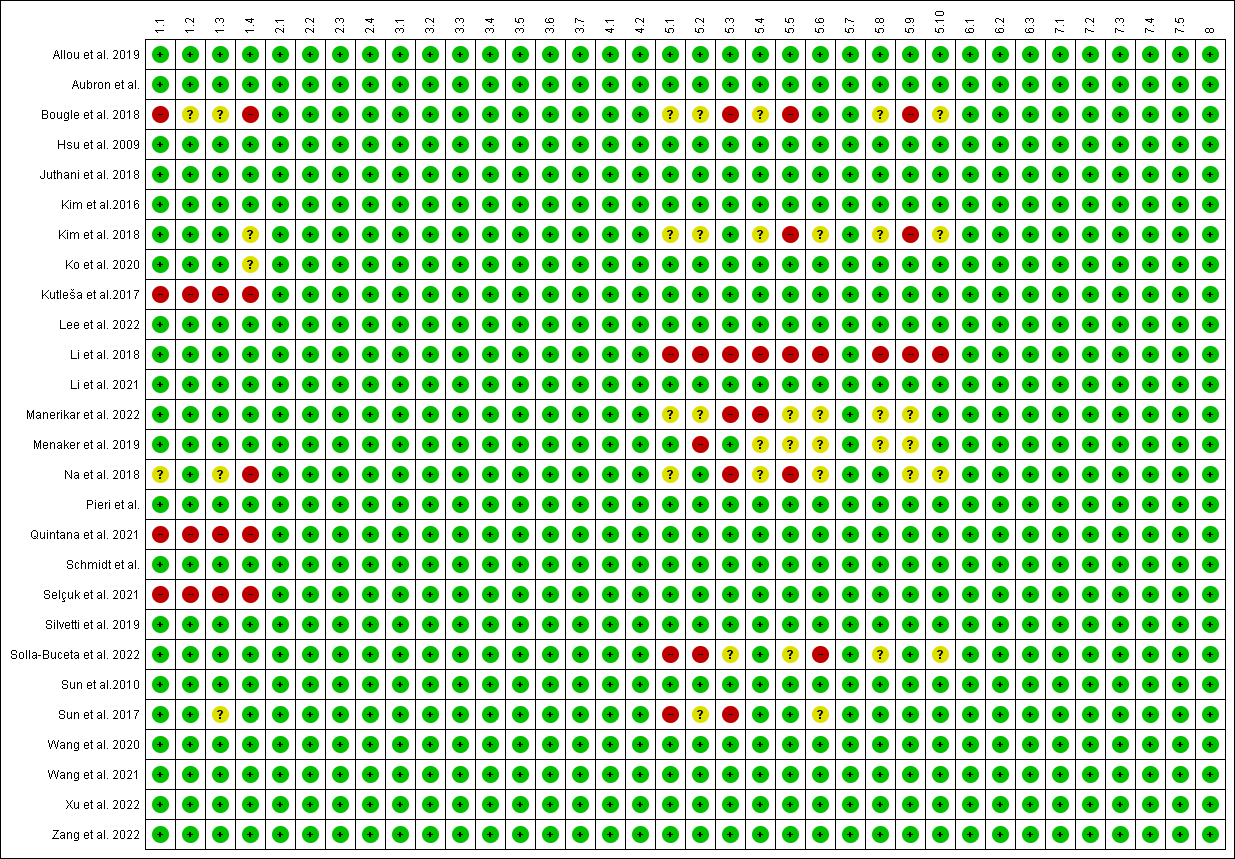


**Table S4:** Risk of bias of included studies based on Cochrane (ROBINS-E) tool according to the categories


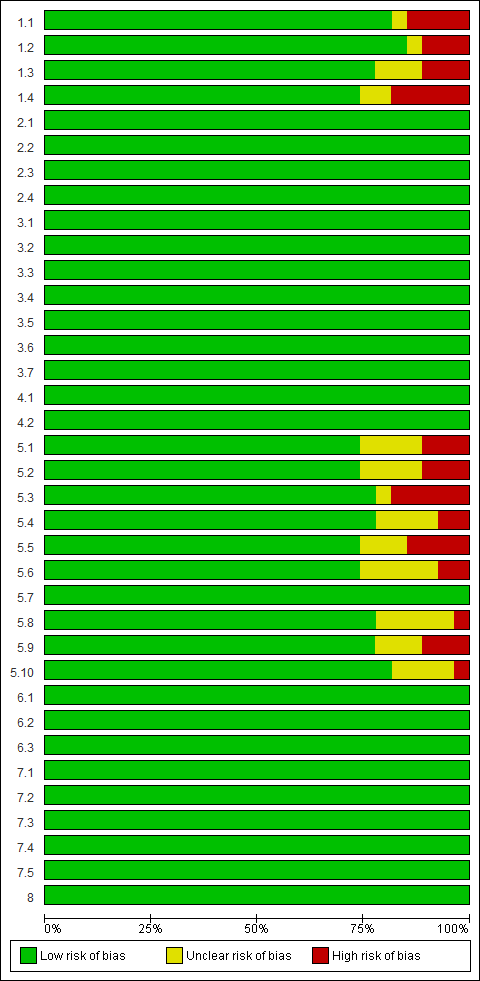


| A |
| --- |
| B |
| C |

**Figure S1:** Compare (A) underlying diseases, (B) severity of illness and (C) length of stay of ECMO, ICU and hospital in patients with and without nosocomial infection based on data of included studies

**Table S5:** ECMO duration, ICU and hospital length of stay in NI and non-NI patients of studies included

|  | ECMO duration | | | ICU length of stay | | | Hospital length of stay | | |
| --- | --- | --- | --- | --- | --- | --- | --- | --- | --- |
|  | **NI group** | **non-NI group** | **P-value** | **NI group** | **non-NI group** | **P-value** | **NI group** | **non-NI group** | **P-value** |
| Hsu et al. [1] | 23.6±24.5 | 7.4±5.8 | 0.001* | 32±30.4 | 17.3±15.3 | 0.010* | 41.9±43.3 | 28.6±33.7 | 0.250 |
| Sun et al. [2] | 360±237 h | 157±163 h | 0.001* | NR | NR | NR | NR | NR | NR |
| Schmidt et al. [3] | 16±17 | 8±5 | 0.001* | 32±25 | 19±21 | 0.004* | 36±30 | 26±29 | 0.010* |
| Aubron et al. [4] | 10 (7-16) | 8 (6-11) | 0.004* | 16 (8-26) | 11 (4-19) | 0.012* | 33.5 (15-55) | 24 (9-42) | 0.029* |
| Pieri et al. [5] | 9 (6-18) | 6 (4-9) | 0.030* | 24.5 (16-55.5) | 12 (8-24) | 0.002* | 27 (18-62) | 16 (11-37) | 0.030* |
| Kim et al. [6] | 17.9±11.5 | 10±7.2 | 0.011* | 51.2±44.2 | 26.6±22 | 0.039* | 68.9±66.5 | 59.2±43.3 | 0.943 |
| Austin et al. [7] | NR | NR | NR | 19 (15-28) | 18 (11-30) | 0.450 | 55 (32-70) | 30 (16-59) | 0.030* |
| Grasselli et al. [8] | 20.5 (10-54) | 10 (5-13) | 0.001* | 32.5 (19-78) | 19 (10-27) | 0.003* | NR | NR | NR |
| kim et al. [9] | 14.5±12.4 | 4.5±2.4 | 0.010* | NR | NR | NR | NR | NR | NR |
| Kutleša et al. [10] | 250 (168-504) h | 150 (102-215) h | 0.001* | NR | NR | NR | NR | NR | NR |
| Sun et al. [11] | NR | NR | NR | NR | NR | NR | 36.5 (25-64) | 7 (7-23) | 0.001* |
| Bougle et al. [12] | NR | NR | NR | NR | NR | NR | NR | NR | NR |
| Juthani et al. [13] | 13 (7-23) | 5 (4-11) | 0.001* | NR | NR | NR | 36.5 (28-48) | 18.5 (10-36) | 0.004* |
| Kim et al. [14] | 14±9.8 | 5.4±1.4 | 0.001* | NR | NR | NR | NR | NR | NR |
| Li et al. [15] | 142.2±140.9 h | 73.8±68.4 h | 0.045* | 298±268.4 h | 172.8±210.7 h | 0.532 | 78±24.4 | 23±20.4 | 0.045* |
| Na et al. [16] | 23 (10-32) | 13 (7-23) | 0.003* | NR | NR | NR | NR | NR | NR |
| Allou et al. [17] | 9 (6-16) | 7 (5-12) | 0.015* | 23 (17-40) | 15 (11-23) | 0.001* | 40 (21-59) | 24 (13-40) | 0.002* |
| Menaker et al. [18] | 18 (13-35) | 9 (5-19) | 0.007* | NR | NR | NR | NR | NR | NR |
| Menaker et al. [18] | 17 (5-20) | 6 (3-9) | 0.020* | NR | NR | NR | NR | NR | NR |
| Silvetti et al. [19] | 432 (83-513) h | 92 (72-168) h | 0.022* | NR | NR | NR | NR | NR | NR |
| Ko et al. [20] | 122 (58-200) h | 71 (45-123) h | 0.006* | 12.8 (5.8-23.3) | 8 (4-18.7) | 0.043* | NR | NR | NR |
| Wang et al. [21] | 276 (152-624) h | 140 (55-727) h | 0.001* | NR | NR | 0.022* | 27.5 (15-40) | 19 (2-42) | 0.025* |
| Wang et al. [22] | 153.5±105.3 h | 105.21±71.79 h | 0.001* | NR | NR | NR | NR | NR | NR |
| Li et al. [23] | 9.5 (5-12) | 4 (3-6) | 0.001* | NR | NR | NR | 55.5 (21-83) | 15.5 (7-23) | 0.001* |
| Quintana et al. [24] | 426.8±383.3 h | 155.5 | 0.001* | NR | NR | NR | NR | NR | NR |
| Quintana et al. [24] | 492.7±383.3 h | 249.1 | 0.004* | NR | NR | NR | NR | NR | NR |
| Selçuk et al. [25] | 11 (1-55) | 2.5 (1-19) | 0.001* | NR | NR | NR | NR | NR | NR |
| Authors names (Ref) | **ECMO duration** | | | **ICU length of stay** | | | **Hospital length of stay** | | |
|  | **NI group** | **non-NI group** | **P-value** | **NI group** | **non-NI group** | **P-value** | **NI group** | **non-NI group** | **P-value** |
| Lee et al. [26] | 13 (7-17.2) | 6.11 (3.8-10.8) | 0.003* | NR | NR | NR | NR | NR | NR |
| Lee et al. [26] | 17.2 (7.5-27.2) | 6.11 (3.8-10.8) | 0.001* | NR | NR | NR | NR | NR | NR |
| Solla-Buceta et al. [27] | 18.8±13.6 | 8.6±7.8 | 0.001* | NR | NR | NR | NR | NR | NR |
| Manerikar et al. [28] | 34±24.8 | 10.4±12.7 | 0.001* | NA | NA | NA | NA | NA | NA |
| Xu et al. [29] | 22±16.5 | 8.9±5.3 | 0.001* | 35.9±22.9 | 15.7±9.2 | 0.001* | NR | NR | NR |
| Zang et al. [30] | 15.97±14.32 | 13.73±14.96 | 0.034* | NR | NR | NR | 35.8±91.9 | 28.6±23.5 | 0.264 |

NI: Nosocomial Infection; NR: Not reported, data expressed as mean ± standard division (SD) or median (IQR), * P<0.05 was considered as significant

**Table S6:** ECMO survival and overall survival in NI and non-NI patients of studies included

| Authors names (Ref) | ECMO survival | | | Overall survival | | |
| --- | --- | --- | --- | --- | --- | --- |
|  | **NI group** | **non-NI group** | **P-value** | **NI group** | **non-NI group** | **P-value** |
| Hsu et al. [1] | 1(10) | 32 (30.8) | 0.277 | 1 (10) | 27 (26) | 0.444 |
| Sun et al. [2] | 19 (42.3) | 179 (61.9) | 0.075 | 11 (24.5) | 95 (32.9) | >0.05 |
| Schmidt et al. [3] | NR | NR | NR | 72 (50.7) | 48 (61.5) | 0.150 |
| Aubron et al. [4] | NR | NR | NR | 21 (58.4) | 75 (68.2) | 0.315 |
| Pieri et al. [5] | 15 (53.6) | 24 (72.2) | 0.200 | 9 (32.1) | 18 (54.5) | 0.989 |
| Kim et al. [6] | NR | NR | NR | 1 (7.7) | 28 (82.4) | 0.001* |
| Austin et al. [7] | 17 (81) | 60 (76.9) | 0.780 | 14 (66.7) | 47 (60.3) | 0.590 |
| Grasselli et al. [8] | NR | NR | NR | NR (59.6) | NR (80) | <0.05* |
| kim et al. [9] | NR | NR | NR | 2 (14.3) | 17 (36.2) | 0.190 |
| Kutleša et al. [10] | NR | NR | NR | 13 (37.14) | 43 (66.1) | NR |
| Sun et al. [11] | NR | NR | NR | NR | NR | NR |
| Bougle et al. [12] | NR | NR | NR | 46 (54.12) | 54 (80.6) | <0.05* |
| Juthani et al. [13] | 22 (84.6) | 58 (78.4) | 0.210 | 13 (50) | 45 (60.8) | 0.210 |
| Kim et al. [14] | NR | NR | NR | NR | NR | NR |
| Li et al. [15] | NR | NR | NR | NR | NR | NR |
| Na et al. [16] | 5 (25) | 60 (60) | 0.003* | 5 (24) | 42 (42) | >0.05 |
| Allou et al. [17] | NR | NR | NR | 20 (51.3) | 96 (53.1) | >0.05 |
| Menaker et al. [18] | NR | NR | NR | 10 (52.6) | 41 (64.3) | >0.05 |
| Menaker et al. [18] | NR | NR | NR | 1 (14.3) | 52 (44.8) | >0.05 |
| Silvetti et al. [19] | 4 (57.1) | 13 (54.2) | 0.617 | 2 (28.6) | 7 (29.2) | 0.681 |
| Ko et al. [20] | 19 (54.3) | 71 (61.7) | 0.554 | 13 (37.1) | 62 (53.9) | 0.209 |
| Wang et al. [21] | NR | NR | NR | 4 (28.6) | 40 (72.7) | 0.004* |
| Wang et al. [22] | 74 (56.5) | 136 (71.2) | 0.012 | 55 (41.98) | 103 (53.9) | 0.001* |
| Li et al. [23] | NR | NR | NR | 15 (93.75) | 34 (85) | NR |
| Quintana et al. [24] | 19 (51.4) | 130 (76.9) | 0.002* | 14 (37.8) | 110 (65.1) | 0.002* |
| Quintana et al. [24] | 14 (77.8) | 50 (87.7) | 0.299 | 12 (66.7) | 42 (73.7) | 0.563 |
| Selçuk et al. [25] | NR | NR | NR | 10 (37.1) | 15 (45.5) | >0.05 |
| Lee et al. [26] | NR | NR | NR | 41 (42.3) | 208 (56.4) | 0.013* |
| Lee et al. [26] | NR | NR | NR | 8 (30.8) | 208 (56.4) | 0.012* |
| Solla-Buceta et al. [27] | NR | NR | NR | NR | NR | NR |
| Manerikar et al. [28] | NR | NR | NR | 4 (26.7) | 31 (67.4) | 0.030* |
| Xu et al. [29] | NR | NR | NR | 12 (28.5) | 13 (35.1) | >0.05 |
| Zang et al. [30] | NR | NR | NR | 35 (92.1) | 133 (85.3) | 0.266 |

NI: Nosocomial Infection; NR: Not reported, data expressed as n (%), *P<0.05 was considered as significant

**Table S7:** Incidence of ECMO-related NI in patients of included studies

| Authors names (Ref) | Time of infection after initiation of ECMO | Total incidence of NI/1000 ECMO-day | Incidence of different types of NI/1000 ECMO-day | | | | | |
| --- | --- | --- | --- | --- | --- | --- | --- | --- |
|  |  |  | **BSI** | **SSI** | **UTI** | **VAP** | **CSI** | **Others** |
| Hsu et al. [1] | 14 days (R: 3-46) | 11.92 | 2.98 | 3.97 | 1.99 | 0 | 0 | 0 |
| Sun et al. [2] | 8 days (R: 2-32) | NR | 14.85 | NR | NR | 0 | 0 | 0 |
| Schmidt et al. [3] | 8 ± 11 | 75.5 | 16 | 0 | 0 | 55.4 | 7.1 | 7.8 |
| Aubron et al. [4] | NR | 30.1 | 15.7 | 0 | NR | NR | 0 | 0 |
| Pieri et al. [5] | NR | 57 | NR | NR | NR | 0 | 0 | NR |
| Kim et al. [6] | NR | NR | NR | 0 | 0 | 0 | 0 | 0 |
| Austin et al. [7] | NR | NR | 8.8 | NR | 0 | 0 | 0 | 0 |
| Grasselli et al. [8] | 9 (4–18.5) | 7.7 | 0 | NR | 31 | NR | NR | NR |
| kim et al. [9] | NR | 43.3 | 21.63 | 0 | 0 | 0 | 0 | 0 |
| Kutleša et al. [10] | NR | NR | NR | NR | NR | NR | NR | NR |
| Sun et al. [11] | NR | NR | NR | 0 | 0 | 0 | 0 | 0 |
| Bougle et al. [12] | 3 (2-8) | NR | 0 | 0 | 0 | 60.6 | 0 | 0 |
| Juthani et al. [13] | NR | 26 | NR | 0 | NR | 0 | 0 | 0 |
| Kim et al. [14] | NR | NR | NR | NR | NR | NR | NR | NR |
| Li et al. [15] | NR | NR | NR | NR | NR | NR | NR | NR |
| Na et al. [16] | 6 days (R:4–19) | 8.5 | 8.5 | 0 | 0 | 0 | 0 | 0 |
| Allou et al. [17] | 9 days (R: 7-14) | 17.2 | NR | 0 | 0 | 0 | NR | 0 |
| Menaker et al. [18] | NR | 8 | 8 | 0 | 0 | 0 | 0 | 0 |
| Menaker et al. [18] | NR | 8 | 8 | 0 | 0 | 0 | 0 | 0 |
| Silvetti et al. [19] | Mean: 7 days | 24.7 | 24.7 | 0 | 0 | 0 | 0 | 0 |
| Ko et al. [20] | Mean: 3 days | 10.7 | 0 | 0 | 0 | 0 | 0 | 0 |
| Wang et al. [21] | NR | 24.7 | 0 | 0 | 0 | 0 | 0 | 0 |
| Wang et al. [22] | NR | 85.4 | NR | NR | NR | 0 | 0 | 0 |
| Li et al. [23] | NR | NR | NR | NR | 0 | 0 | 0 | NR |
| Quintana et al. [24] | 6.2±3.7 days | 15.5 | 6.8 | 0 | 0 | 0 | 0 | 0 |
| Quintana et al. [24] | 19±11.8 days | 17.3 | 9.3 | 0 | 0 | 0 | 0 | 0 |
| Selçuk et al. [25] | 2.70±3.72 | 57 | 4 | 17 | 4 | 0 | 11 | 0 |
| Lee et al. [26] | 8 days (R: 5-16) | 17.9 | 17.9 | 0 | 0 | 0 | 0 | 0 |
| Lee et al. [26] | 11 days (R: 6-20) | 4.1 | 4.1 | 0 | 0 | 0 | 0 | 0 |
| Solla-Buceta et al. [27] | NR | 33.8 | NR | NR | NR | NR | NR | NR |
| Manerikar et al. [28] | Mean: 15.4 days | NR | NR | 0 | 0 | 0 | 0 | 0 |
| Xu et al. [29] | NR | NR | 15.2 | 0 | 3.2 | 23.9 | 0 | 0 |
| Zang et al. [30] | NR | NR | NR | NR | NR | NR | NR | NR |

Abbreviations: blood stream infection (BSI), urinary tract infection (UTI), surgical site infection (SSI), cannula site infection (CSI), nosocomial Infection (NI), and not reported (NR)

**Table S8:** Micro-organisms classification

**1. Bacteria**

***1.1 Gram negative***

- ***E. coli****:* A Gram-negative
- ***Acinetobacter baumannii***: A Gram-negative
- ***Pseudomonas aeruginosa***: A Gram-negative
- ***Klebsiella pneumonia****:* A Gram-negative
- ***Klebsiella oxytoca****:* A Gram-negative
- ***Enterobacter cloacae****:* A Gram-negative
- ***Enterobacter aeruginosa****:* A Gram-negative
- ***Stenotrophomonas maltophilia****:* A Gram-negative
- ***Serratia spp****.:* A Gram-negative
- ***Burkholderia cepacia****:* A Gram-negative
- ***Neisseria spp****.:* Neisseria spp. are part of the commensal flora of mucosal membranes of humans and some animals, and are generally considered non-pathogenic except for N. gonorrhoea and N. meningitides
- ***Proteus mirabilis***: A Gram-negative
- ***Aeromonas caviae****:* A Gram-negative
- ***Bacteroides ssp****.:* A Gram-negative
- ***Citrobacter spp***: A Gram-negative

***1.2. Gram positive***

- ***Enterococcus ssp****.:* A Gram-Positive
- ***Streptococcus spp****.:* A Gram-Positive
- ***MRSA****:* A Gram-Positive
- ***VRE****:* A Gram-Positive
- ***Staphylococcus epidermidis***: A Gram-Positive
- ***Staphylococcus aureus***: A Gram-Positive
- ***Anaerobes spp****.:* A Gram-Positive
- ***Coagulase-negative staphylococci****:* A Gram-Positive

***1.3 multiple or not define***

Polymicrobial: The rate of polymicrobial infections, or infections involving two or more micro-organisms.

**2. Fungus**

- ***Candida spp****.* Means we don’t have the specific species
- ***Yeast***: it is a yeast but not defined
- ***Aspergillus spp****.:* Aspergillosis is an infection caused by Aspergillus, a common mold (a type of fungus) that lives indoors and outdoors. Most people breathe in Aspergillus spores every day without getting sick.

**3. Viral**

**Influenza** … it can be virus or if haemophilus influenza which is a bacterium

**Table S9:** Number of pathogens isolated from each episode NI in studies included

| Authors names (Ref) | No. of  pathogen isolated | Microorganisms | | | | | |
| --- | --- | --- | --- | --- | --- | --- | --- |
|  |  | **GNB** | **GPB** | **Fungus** | **Influenza** | **Poly-microbial** | **Others** |
| Hsu et al. [1] | 18 | 14 (77.8) | 3 (16.7) | 1 (5.6) | 0 | 0 | 0 |
| Sun et al. [2] | 66 | 43 (65.1) | 11 (16.7) | 10 (15.1) | 0 | 0 | 2 (3.03) |
| Schmidt et al. [3] | 254 | 135 (53.1) | 65 (25.6) | 14 (5.5) | 14 (5.5) | 23 (9.1) | 3 (1.2) |
| Aubron et al. [4] | 53 | 26 (49.1) | 11 (20.7) | 9 (16.9) | 0 | 0 | 7 (13.2) |
| Pieri et al. [5] | 56 | 26 (46.4) | 11 (19.6) | 15 (26.8) | 0 | 0 | 4 (7.1) |
| Kim et al. [6] | 13 | 7 (53.8) | 4 (30.7) | 2 (15.4) | 0 | 0 | 0 |
| Austin et al. [7] | 29 | 15 (51.7) | 5 (17.2) | 8 (27.6) | 0 | 0 | 1 (3.4) |
| Grasselli et al. [8] | 52 | 25 (48.1) | 15 (28.8) | 9 (17.3) | 0 | 0 | 3 (5.8) |
| kim et al. [9] | 18 | 14 (77.8) | 2 (11.1) | 1 (5.6) | 0 | 0 | 1 (5.6) |
| Kutleša et al. [10] | 36 | 21 (58.3) | 12 (33.3) | 1 (2.8) | 0 | 2 (5.6) | 0 |
| Sun et al. [11] | 58 | 43 (74.1) | 15 (25.8) | 0 | 0 | 0 | 0 |
| Bougle et al. [12] | 121 | 71 (58.7) | 10 (8.3) | 4 (3.3) | 7 (5.8) | 0 | 29 (23.9) |
| Juthani et al. [13] | 26 | NR | NR | NR | NR | NR | NR |
| Kim et al. [14] | NR | NR | NR | NR | NR | NR | NR |
| Li et al. [15] | 38 | 17 (44.7) | 14 (36.8) | 1 (2.6) | 0 | 0 | 6 (15.8) |
| Na et al. [16] | NR | NR | NR | NR | NR | NR | NR |
| Allou et al. [17] | 71 | 40 (56.3) | 27 (38.1) | 1 (1.4) | 0 | 0 | 3 (4.2) |
| Menaker et al. [18] | 19 | 5 (26.3) | 7 (36.8) | 5 (26.3) | 0 | 2 (10.5) | 0 |
| Menaker et al. [18] | 7 | 5 (71.4) | 0 | 1 (14.3) | 0 | 1 (14.3) | 0 |
| Silvetti et al. [19] | 7 | NR | NR | NR | NR | NR | NR |
| Ko et al. [20] | 35 | 20 (57.1) | 12 (34.3) | 2 (5.7) | 0 | 0 | 1 (2.9) |
| Wang et al. [21] | 14 | 14 (100) | 0 | 0 | 0 | 0 | 0 |
| Wang et al. [22] | 487 | 191 (39.2) | 57 (11.7) | 86 (17.7) | 0 | 0 | 153 (31.4) |
| Li et al. [23] | 53 | 32 (60.4) | 0 | 8 (15.1) | 0 | 0 | 13 (24.5) |
| Quintana et al. [24] | 30 | 11 (36.7) | 9 (30.0) | 8 (26.7) | 0 | 0 | 2 (6.7) |
| Quintana et al. [24] | 19 | 7 (36.8) | 7 (36.8) | 3 (15.8) | 0 | 0 | 2 (10.5) |
| Selçuk et al. [25] | 27 | 18 (66.7) | 5 (18.5) | 0 | 0 | 0 | 4 (14.8) |
| Lee et al. [26] | 153 | 52 (33.9) | 69 (45.1) | 28 (18.3) | 0 | 0 | 4 (2.61) |
| Lee et al. [26] | 153 | 52 (33.9) | 69 (45.1) | 28 (18.3) | 0 | 0 | 4 (2.61) |
| Solla-Buceta et al. [27] | 100 | 49 (49.0) | 27 (27.0) | 10 (10.0) | 0 | 0 | 14 (14.0) |
| Manerikar et al. [28] | 19 | 5 (26.3) | 9 (47.4) | 5 (26.3) | 0 | 0 | 0 |
| Xu et al. [29] | 53 | 42 (79.2) | 4 (7.5) | 3 (5.6) | 0 | 4 (7.5) | 0 |
| Zang et al. [30] | NR | NR | NR | NR | NR | NR | NR |

GNB: gram negative bacteria, GPB: gram positive bacteria

| **A**   |
| --- |
| **B**   |

**Figure S2:** Forest plot showed adjusted binary logistic regression analysis to predict developed NI by (A) ECMO (based on days or hours) duration in the 17 included studies between patients with and without NI and (B) demographic and clinical variables in studies included. Abbreviations; ECPR: Extracorporeal cardiopulmonary resuscitation, BMI: body mass index, ECMO: extracorporeal membrane oxygenation, SAPS: simplified acute physiology score, SOFA: sequential organ failure assessment score, VA: veno-arterial ECMO, VV: veno-venous ECMO, ICU: intensive care units, LOS: length of stay, MV: mechanical ventilator, mechanical complications included oxygenator failure; pump malfunction; heater dysfunction; clot of oxygenator, hemofilter, and other sites, Pulmonary complications included pneumothorax and pulmonary hemorrhage.

**Figure S3:** Forest plot for pooled risk ratio of ECMO duration to predict nosocomial infection (NI)

**Table S10:** Meta-regression analysis for exploring heterogeneity among studies

| Variables | Coefficient | P-Value | 95% CI |
| --- | --- | --- | --- |
| ECMO survival for all participants | | | |
| APACHEII IP | - 0.140 | 0.627 | -0.701 to -0.423 |
| APACHE II NIP | - 0.311 | 0.003* | -0.518 to -0.103 |
| ECMO cause (Respiratory) IP | 0.089 | 0.359 | -0.101 to 0.278 |
| ECMO cause (Respiratory) NIP | 0.143 | 0.091* | -0.023 to 0.310 |
| ECMO cause (Cardiogenic) IP | - 0.089 | 0.359 | -0.278 to 0.101 |
| ECMO cause (Cardiogenic) NIP | - 0.152 | 0.058* | -0.310 to 0.005 |
| VA ECMO IP | - 0.088 | 0.348 | -0.271 to 0.096 |
| VA ECMO NIP | - 0.154 | 0.073* | -0.323 to -0.015 |
| ECMO survival between infected and non-infected patients | | | |
| VA ECMO IP | - 0.008 | 0.982 | -0.408 to 0.398 |
| VA ECMO NIP | - 0.352 | 0.001* | -0.558 to -0.145 |
| Number of isolated pathogens | - 0.280 | 0.071* | -0.585 to -0.024 |
| Hospital discharge survival for all participants | | | |
| Age IP | - 0.060 | 0.274 | -0.168 to 0.048 |
| Age NIP | - 0.176 | <0.001* | -0.267 to -0.085 |
| COPD IP | 0.159 | 0.012* | 0.035 to 0.283 |
| COPD NIP | 0.047 | 0.546 | -0.107 to 0.201 |
| ESRD IP | 0.134 | 0.114 | -0.032 to 0.301 |
| ESRD NIP | 0.193 | 0.013* | 0.040 to 0.347 |
| ECMO cause (Cardiogenic) IP | - 0.087 | 0.135 | -0.202 to 0.027 |
| ECMO cause (Cardiogenic) NIP | - 0.128 | 0.029* | -0.243 to -0.013 |
| ECMO duration IP | - 0.036 | 0.600 | -0.170 to 0.098 |
| ECMO duration NIP | - 0.135 | 0.051* | -0.0005 to 0.271 |
| Hospital discharge survival between infected and non-infected patients | | | |
| Age IP | - 0.157 | 0.169 | -0.380 to 0.067 |
| Age NIP | - 0.211 | 0.041* | -0.422 to 0.0008 |
| HTN IP | - 0.346 | <0.001* | -0.506 to -0.186 |
| HTN NIP | - 0.391 | <0.001* | -0.560 to -0.221 |
| In-hospital mortality for all participants | | | |
| Age IP | 0.064 | 0.250 | -0.045 to 0.174 |
| Age NIP | 0.170 | <0.001* | 0.076 to 0.264 |
| COPD IP | - 0.159 | 0.012* | -0.283 to -0.035 |
| COPD NIP | - 0.047 | 0.546 | -0.202 to 0.107 |
| ESRD IP | - 0.143 | 0.096* | -0.312 to 0.026 |
| ESRD NIP | - 0.202 | 0.010* | -0.357 to -0.048 |
| ECMO cause (Cardiogenic) IP | 0.083 | 0.162 | -0.034 to 0.200 |
| ECMO cause (Cardiogenic) NIP | 0.124 | 0.038* | 0.007 to 0.242 |
| ECMO duration IP | 0.039 | 0.572 | -0.097 to 0.176 |
| ECMO duration NIP | 0.137 | 0.051* | -0.275 to 0.0005 |
| In-hospital mortality between infected and non-infected patients | | | |
| HTN IP | - 0.021 | 0.867 | -0.272 to 0.229 |
| HTN NIP | 0.178 | 0.062* | -0.009 to 0.364 |
| ESRD IP | 0.119 | 0.232 | -0.077 to 0.316 |
| ESRD NIP | 0.199 | 0.063* | -0.011 to 0.408 |
| ECMO cause (Respiratory) IP | 0.043 | 0.608 | -0.122 to 0.208 |
| ECMO cause (Respiratory) NIP | 0.134 | 0.055* | -0.003 to 0.271 |

*P<0.1 considered as significant, IP: infected patients, NIP: n0n-infected patients, APACHE II: Acute Physiology and Chronic Health Evaluation II, VA: veno-arterial ECMO, VV: veno-venous ECMO, HTN: hypertension, COPD: chronic pulmonary diseases, CAD: coronary artery diseases, ESRD: end-stage renal diseases

| **A**   |
| --- |
| **B**   |

**Figure S4:**  Cumulative analysis of ECMO survival for (A) all participants in each study, (B) between infected and non-infected patients

| **A**   |
| --- |
| **B**   |

**Figure S5:**  Cumulative analysis of overall survival for (A) all participants in each study, (B) between infected and non-infected patients

**References**

1. Hsu MS, Chiu KM, Huang YT, Kao KL, Chu SH, Liao CH. Risk factors for nosocomial infection during extracorporeal membrane oxygenation. J Hosp Infect. 2009, 73(3):210-216. <https://doi.org/10.1016/j.jhin.2009.07.016>

2. Sun HY, Ko WJ, Tsai PR, Sun CC, Chang YY, Lee CW*, et al*. Infections occurring during extracorporeal membrane oxygenation use in adult patients. J Thorac Cardiovasc Surg. 2010, 140(5):1125-1132.e1122. <https://doi.org/10.1016/j.jtcvs.2010.07.017>

3. Schmidt M, Brechot N, Hariri S, Guiguet M, Luyt CE, Makri R*, et al*. Nosocomial Infections in Adult Cardiogenic Shock Patients Supported by Venoarterial Extracorporeal Membrane Oxygenation. Clin Infect Dis. 2012, 55(12):1633-1641. <https://doi.org/10.1093/cid/cis783>

4. Aubron C, Cheng AC, Pilcher D, Leong T, Magrin G, Cooper DJ*, et al*. Infections acquired by adults who receive extracorporeal membrane oxygenation: risk factors and outcome. Infection control and hospital epidemiology. 2013, 34(1):24-30. <https://doi.org/10.1086/668439>

5. Pieri M, Agracheva N, Fumagalli L, Greco T, De Bonis M, Calabrese MC*, et al*. Infections occurring in adult patients receiving mechanical circulatory support: The two-year experience of an italian national referral tertiary care center. Med Intensiva. 2013, 37(7):468-475. <https://doi.org/10.1016/j.medin.2012.08.009>

6. Kim DW, Yeo HJ, Yoon SH, Lee SE, Lee SJ, Cho WH*, et al*. Impact of bloodstream infections on catheter colonization during extracorporeal membrane oxygenation. J Artif Organs. 2016, 19(2):128-133. <https://doi.org/10.1007/s10047-015-0882-5>

7. Austin DE, Kerr SJ, Al-Soufi S, Connellan M, Spratt P, Goeman E*, et al*. Nosocomial infections acquired by patients treated with extracorporeal membrane oxygenation. Crit Care Resusc. 2017, 19:68-75.

8. Grasselli G, Scaravilli V, Di Bella S, Biffi S, Bombino M, Patroniti N*, et al*. Nosocomial infections during extracorporeal membrane oxygenation: Incidence, etiology, and impact on patients' outcome. Crit Care Med. 2017, 45(10):1726-1733. <https://doi.org/10.1097/CCM.0000000000002652>

9. Kim GS, Lee KS, Park CK, Kang SK, Kim DW, Oh SG*, et al*. Nosocomial infection in adult patients undergoing veno-arterial extracorporeal membrane oxygenation. J Korean Med Sci. 2017, 32(4):593-598. <https://doi.org/10.3346/jkms.2017.32.4.593>

10. Kutleša M, Santini M, Krajinović V, Papić N, Novokmet A, Josipović Mraović R*, et al*. Nosocomial blood stream infections in patients treated with venovenous extracorporeal membrane oxygenation for acute respiratory distress syndrome. Minerva Anestesiol. 2017, 83(5):493-501. <https://doi.org/10.23736/S0375-9393.17.11659-7>

11. Sun G, Li B, Lan H, Wang J, Lu L, Feng X*, et al*. Risk factors for nosocomial infections in patients receiving extracorporeal membrane oxygenation supportive therapy. Med Clin. 2017, 149(10):423-428. <https://doi.org/10.1016/j.medcli.2017.03.038>

12. Bougle A, Bombled C, Margetis D, Lebreton G, Vidal C, Coroir M*, et al*. Ventilator-associated pneumonia in patients assisted by veno-arterial extracorporeal membrane oxygenation support: Epidemiology and risk factors of treatment failure. Plos One. 2018, 13(4). <https://doi.org/10.1371/journal.pone.0194976>

13. Juthani BK, Macfarlan J, Wu J, Misselbeck TS. Incidence of nosocomial infections in adult patients undergoing extracorporeal membrane oxygenation. Heart Lung. 2018, 47(6):626-630. <https://doi.org/10.1016/j.hrtlng.2018.07.004>

14. Kim DW, Cho HJ, Kim GS, Song SY, Na KJ, Oh SG*, et al*. Predictive Value of Procalcitonin for Infection and Survival in Adult Cardiogenic Shock Patients Treated with Extracorporeal Membrane Oxygenation. Chonnam medical journal. 2018, 54(1):48-54. <https://doi.org/10.4068/cmj.2018.54.1.48>

15. Li B, Sun G, Cheng Z, Mei C, Liao X, Li J*, et al*. Analysis of nosocomial infections in post-cardiac surgery extracorporeal membrane oxygenation support therapy. Heart Surg Forum. 2018, 21(5):E387-E391. <https://doi.org/10.1532/hsf.1789>

16. Na SJ, Chung CR, Choi HJ, Cho YH, Yang JH, Suh GY*, et al*. Blood Stream Infection in Patients on Venovenous Extracorporeal Membrane Oxygenation for Respiratory Failure. Infection control and hospital epidemiology. 2018, 39(7):871-874. <https://doi.org/10.1017/ice.2018.90>

17. Allou N, Lo Pinto H, Persichini R, Bouchet B, Braunberger E, Lugagne N*, et al*. Cannula-related infection in patients supported by peripheral ECMO: Clinical and microbiological characteristics. ASAIO J. 2019, 65(2):180-186. <https://doi.org/10.1097/MAT.0000000000000771>

18. Menaker J, Galvagno S, Rabinowitz R, Penchev V, Hollis A, Kon Z*, et al*. Epidemiology of blood stream infection in adult extracorporeal membrane oxygenation patients: A cohort study. Heart Lung. 2019, 48(3):236-239. <https://doi.org/10.1016/j.hrtlng.2019.01.004>

19. Silvetti S, Ranucci M, Pistuddi V, Isgro G, Ballotta A, Ferraris L*, et al*. Bloodstream infections during post-cardiotomy extracorporeal membrane oxygenation: Incidence, risk factors, and outcomes. Int J Artif Organs. 2019, 42(6):299-306. <https://doi.org/10.1177/0391398818817325>

20. Ko RE, Huh K, Kim DH, Na SJ, Chung CR, Cho YH*, et al*. Nosocomial infections in in-hospital cardiac arrest patients who undergo extracorporeal cardiopulmonary resuscitation. PLoS ONE. 2020, 15(12 December). <https://doi.org/10.1371/journal.pone.0243838>

21. Wang J, Huang J, Hu W, Cai X, Hu W, Zhu Y. Risk factors and prognosis of nosocomial pneumonia in patients undergoing extracorporeal membrane oxygenation: a retrospective study. J Int Med Res. 2020, 48(10). <https://doi.org/10.1177/0300060520964701>

22. Wang J, Wang L, Jia M, Du Z, Hou X. Extracorporeal membrane oxygenation-related nosocomial infection after cardiac surgery in adult patients. Braz J Cardiovasc Surg. 2021, 36(6):743-751. <https://doi.org/10.21470/1678-9741-2020-0068>

23. Li ZJ, Zhang DF, Zhang WH. Analysis of nosocomial infection and risk factors in patients with ECMO treatment. Infect Drug Resist. 2021, 14:2403-2410. <https://doi.org/10.2147/IDR.S306209>

24. Quintana MT, Mazzeffi M, Galvagno SM, Herrera D, Boyajian GP, Hays NM*, et al*. A Retrospective Study of Infection in Patients Requiring Extracorporeal Membrane Oxygenation Support. Ann Thorac Surg. 2021, 112(4):1168-1175. <https://doi.org/10.1016/j.athoracsur.2020.12.012>

25. Selçuk Ü N, Sargın M, Baştopçu M, Mete EMT, Erdoğan SB, Öcalmaz Ş*, et al*. Microbiological Spectrum of Nosocomial ECMO Infections in a Tertiary Care Center. Braz J Cardiovasc Surg. 2021, 36(3):338-345. <https://doi.org/10.21470/1678-9741-2020-0077>

26. Lee EH, Lee KH, Lee SJ, Kim J, Baek YJ, Ahn JY*, et al*. Clinical and microbiological characteristics of and risk factors for bloodstream infections among patients with extracorporeal membrane oxygenation: a single-center retrospective cohort study. Sci Rep. 2022, 12(1). <https://doi.org/10.1038/s41598-022-19405-z>

27. Solla-Buceta M, Gonzalez-Vilchez F, Almenar-Bonet L, Lambert-Rodriguez JL, Segovia-Cubero J, Gonzalez-Costello J*, et al*. Infectious complications associated with short-term mechanical circulatory support in urgent heart transplant candidates. Rev Esp Cardiol. 2022, 75(2):141-149. <https://doi.org/10.1016/j.rec.2020.11.019>

28. Manerikar A, Watanabe S, Kandula V, Karim A, Thakkar S, Saine M*, et al*. Indwelling Central Venous Catheters Drive Bloodstream Infection During Veno-venous Extracorporeal Membrane Oxygenation Support. ASAIO J. 2022, 68(6):859-864. <https://doi.org/10.1097/MAT.0000000000001575>

29. Xu W, Fu Y, Yao Y, Zhou J, Zhou H. Nosocomial Infections in Nonsurgical Patients Undergoing Extracorporeal Membrane Oxygenation: A Retrospective Analysis in a Chinese Hospital. Infect Drug Resist. 2022, 15:4117-4126. <https://doi.org/10.2147/IDR.S372913>

30. Zang F, Zhang X, Liu J, Li S, Zhang Y, Li Z. Hospital expenses of nosocomial infection associated with extracorporeal membrane oxygenation in China: a retrospective cohort study. Ann Palliat Med. 2022, 11(2):431-441. <https://doi.org/10.21037/apm-21-1825>
