# Supplementary material for: Incidence, risk factors and outcomes of nosocomial infection in adult patients supported by extracorporeal membrane oxygenation: a systematic review and meta-analysis
Source: Crit Care. 2024 May 10;28:158. doi: 10.1186/s13054-024-04946-8 (PMC11088079; doi:10.1186/s13054-024-04946-8)
Supplement: Supplementary file 3 — Additional file 3 (DOCX 217 KB) [file 13054_2024_4946_MOESM3_ESM.docx]

***Subgroup analysis***

The subgroup analysis revealed significant differences in ECMO survival and overall survival rates across different countries. Specifically, in China and Taiwan, ECMO survival rates were lower in infected patients compared to non-infected patients, ranging from 0.65 to 0.79. In other countries such as Italy, Australia, Korea, and the USA, ECMO survival rates ranged from 0.45 to 0.78. Overall survival rates varied widely among countries, with China reporting the highest rate (65%) and Taiwan the lowest (30%). Interestingly, infected patients had significantly lower overall survival rates compared to non-infected patients in Croatia, Korea, and France. These findings imply that the impact of infection on ECMO and overall survival rates can differ across countries, highlighting the need for further investigation (Figures 1A-1D).

***Sensitivity analysis***

To identify the source of heterogeneity among studies, a sensitivity analysis was conducted. This involved excluding specific studies to assess their impact on the pooled estimate. For the incidence analysis, the five studies [1-5] that had the highest influence on the overall incidence index were identified. After removing these studies to reduce heterogeneity, the adjusted analysis revealed a pooled incidence of NI per 1000 ECMO-day (based on 13 studies and 2761 patients) of 0.12 (95% CI: 0.07-0.16, P<0.001), with mild significant heterogeneity (I^2^=35.7%, P=0.01) (Figure 5B). A sensitivity analysis was also performed for studies examining ECMO survival and overall survival in all participants, as well as between infected and non-infected patients. It was determined that no study was affected by heterogeneity (Figures 2A/2B and 3A/3B). Furthermore, the Galbraith plot was used to assess heterogeneity for ECMO survival and overall survival (Figures 4A-4D), which indicated the absence of heterogeneity.

***Publication bias***

Upon examining the funnel plot for ECMO survival in all patients and conducting the Egger (P=0.985) and Begg (P=0.631) tests (Figure 5A), no evidence of publication bias was found. The trim-and-fill method also aligned with the funnel plot. However, for ECMO survival between infected and non-infected patients, the funnel plot displayed slight asymmetry (Figure 5B), and the Egger test indicated publication bias (P=0.006), while the Begg test did not show publication bias (P=0.086). The funnel plot for overall survival in all patients demonstrated slight asymmetry (Figure 5C), but neither the Egger (P=0.904) nor the Begg (P=0.957) tests detected any publication bias. The trim-and-fill method further confirmed the absence of publication bias. However, the funnel plot for overall survival between infected and non-infected patients revealed publication bias (Figure 5D), which was supported by the Egger test (P=0.003), but not by the Begg test (P=0.101).

| **A**   | **B**   |
| --- | --- |
| **C**   | **D**   |

**Figure 1:** Forest plot of ECMO survival and overall survival for (A and C) all participants in each study and (B and D) between infected and non-infected patients, according to countries subgroup. "The results of several studies indicate that ECMO survival rates vary depending on the country and number of participants. In Taiwan, two separate studies with a total of 448 patients reported an RR of 0.45 (95%CI: 0.15-0.75) [6, 7]. Italy had two studies with 92 patients and an RR of 0.61 (95%CI: 0.41-0.81) [2, 8], while Australia had one study with 99 patients and an RR of 0.78 (95%CI: 0.58-0.97) [9], Korea had three studies with 309 patients and an RR of 0.52 (95%CI: 0.41-0.63) [10-12], and the USA had two studies with 381 patients and an RR of 0.77 (95%CI: 0.67-0.87) [13, 14]. Additionally, China had one study with 322 patients and an RR of 0.65 (95%CI: 0.54-0.76) [15] (A). However, when comparing infected patients to non-infected patients, both China (RR: 0.79, 95%CI: 0.67-0.95) and Taiwan (RR: 0.66, 95%CI: 0.47-0.94) had significantly lower ECMO survival rates (B). Looking at overall survival rates in infected and non-infected patients across 26 studies from various countries (including Taiwan, France, Australia, Italy, Korea, USA, China, Turkey, and Croatia), there was a significant difference in Croatia (RR: 0.58, 95%CI: 0.25-0.89), Korea (RR: 0.67, 95%CI: 0.55-0.82), and France (RR: 0.79, 95%CI: 0.65-0.98) between infected and non-infected patients, with Taiwan having the lowest overall survival rate at 30% (with a range of 21%-39%) and China having the highest overall survival rate at 65% (95%CI: 48%-84%) (C and D)."

| **A**   |
| --- |
| **B**   |

**Figure 2:**  Sensitivity analysis of ECMO survival for (A) all participants in each study, (B) between infected and non-infected patients

| **A**   |
| --- |
| **B**   |

**Figure 3:**  Sensitivity analysis of overall survival for (A) all participants in each study, (B) between infected and non-infected patients

| **A**   | **B**   |
| --- | --- |
| **C**   | **D**   |

**Figure 4**: Galbraith plot of ECMO survival for (A) all participants in each study, (B) between infected and non-infected patients and for overall survival (C) all participants in each study, (D) between infected and non-infected patients

| **A**   | **B**   |
| --- | --- |
| **C**   | **D**   |

**Figure 5:** Funnel plot of ECMO survival for (A) all participants in each study, (B) between infected and non-infected patients and overall survival for (C) all participants in each study, (D) between infected and non-infected patients

**References**

1. Schmidt M, Brechot N, Hariri S, Guiguet M, Luyt CE, Makri R*, et al*. Nosocomial Infections in Adult Cardiogenic Shock Patients Supported by Venoarterial Extracorporeal Membrane Oxygenation. Clin Infect Dis. 2012, 55(12):1633-1641. <https://doi.org/10.1093/cid/cis783>

2. Pieri M, Agracheva N, Fumagalli L, Greco T, De Bonis M, Calabrese MC*, et al*. Infections occurring in adult patients receiving mechanical circulatory support: The two-year experience of an italian national referral tertiary care center. Med Intensiva. 2013, 37(7):468-475. <https://doi.org/10.1016/j.medin.2012.08.009>

3. Kim GS, Lee KS, Park CK, Kang SK, Kim DW, Oh SG*, et al*. Nosocomial infection in adult patients undergoing veno-arterial extracorporeal membrane oxygenation. J Korean Med Sci. 2017, 32(4):593-598. <https://doi.org/10.3346/jkms.2017.32.4.593>

4. Wang J, Wang L, Jia M, Du Z, Hou X. Extracorporeal membrane oxygenation-related nosocomial infection after cardiac surgery in adult patients. Braz J Cardiovasc Surg. 2021, 36(6):743-751. <https://doi.org/10.21470/1678-9741-2020-0068>

5. Selçuk Ü N, Sargın M, Baştopçu M, Mete EMT, Erdoğan SB, Öcalmaz Ş*, et al*. Microbiological Spectrum of Nosocomial ECMO Infections in a Tertiary Care Center. Braz J Cardiovasc Surg. 2021, 36(3):338-345. <https://doi.org/10.21470/1678-9741-2020-0077>

6. Hsu MS, Chiu KM, Huang YT, Kao KL, Chu SH, Liao CH. Risk factors for nosocomial infection during extracorporeal membrane oxygenation. J Hosp Infect. 2009, 73(3):210-216. <https://doi.org/10.1016/j.jhin.2009.07.016>

7. Sun HY, Ko WJ, Tsai PR, Sun CC, Chang YY, Lee CW*, et al*. Infections occurring during extracorporeal membrane oxygenation use in adult patients. J Thorac Cardiovasc Surg. 2010, 140(5):1125-1132.e1122. <https://doi.org/10.1016/j.jtcvs.2010.07.017>

8. Silvetti S, Ranucci M, Pistuddi V, Isgro G, Ballotta A, Ferraris L*, et al*. Bloodstream infections during post-cardiotomy extracorporeal membrane oxygenation: Incidence, risk factors, and outcomes. Int J Artif Organs. 2019, 42(6):299-306. <https://doi.org/10.1177/0391398818817325>

9. Austin DE, Kerr SJ, Al-Soufi S, Connellan M, Spratt P, Goeman E*, et al*. Nosocomial infections acquired by patients treated with extracorporeal membrane oxygenation. Crit Care Resusc. 2017, 19:68-75.

10. Kim DW, Cho HJ, Kim GS, Song SY, Na KJ, Oh SG*, et al*. Predictive Value of Procalcitonin for Infection and Survival in Adult Cardiogenic Shock Patients Treated with Extracorporeal Membrane Oxygenation. Chonnam medical journal. 2018, 54(1):48-54. <https://doi.org/10.4068/cmj.2018.54.1.48>

11. Ko RE, Huh K, Kim DH, Na SJ, Chung CR, Cho YH*, et al*. Nosocomial infections in in-hospital cardiac arrest patients who undergo extracorporeal cardiopulmonary resuscitation. PLoS ONE. 2020, 15(12 December). <https://doi.org/10.1371/journal.pone.0243838>

12. Na SJ, Chung CR, Choi HJ, Cho YH, Yang JH, Suh GY*, et al*. Blood Stream Infection in Patients on Venovenous Extracorporeal Membrane Oxygenation for Respiratory Failure. Infection control and hospital epidemiology. 2018, 39(7):871-874. <https://doi.org/10.1017/ice.2018.90>

13. Juthani BK, Macfarlan J, Wu J, Misselbeck TS. Incidence of nosocomial infections in adult patients undergoing extracorporeal membrane oxygenation. Heart Lung. 2018, 47(6):626-630. <https://doi.org/10.1016/j.hrtlng.2018.07.004>

14. Quintana MT, Mazzeffi M, Galvagno SM, Herrera D, Boyajian GP, Hays NM*, et al*. A Retrospective Study of Infection in Patients Requiring Extracorporeal Membrane Oxygenation Support. Ann Thorac Surg. 2021, 112(4):1168-1175. <https://doi.org/10.1016/j.athoracsur.2020.12.012>

15. Wang WW, Zhou QX, Ma L, Feng SH, Yang ZR, Sun F*, et al*. [Introduction of a tool to assess Risk of Bias in Non-randomized Studies-of Environmental Exposure (ROBINS-E)]. Zhonghua Liu Xing Bing Xue Za Zhi. 2022, 43(1):98-104. <https://doi.org/10.3760/cma.j.cn112338-20201112-01324>
