# Supplementary material for: Incidence, risk factors and outcomes of nosocomial infection in adult patients supported by extracorporeal membrane oxygenation: a systematic review and meta-analysis
Source: Crit Care. 2024 May 10;28:158. doi: 10.1186/s13054-024-04946-8 (PMC11088079; doi:10.1186/s13054-024-04946-8)
Supplement: Supplementary file 4 — Additional file 4 (DOCX 2245 KB) [file 13054_2024_4946_MOESM4_ESM.docx]

| A  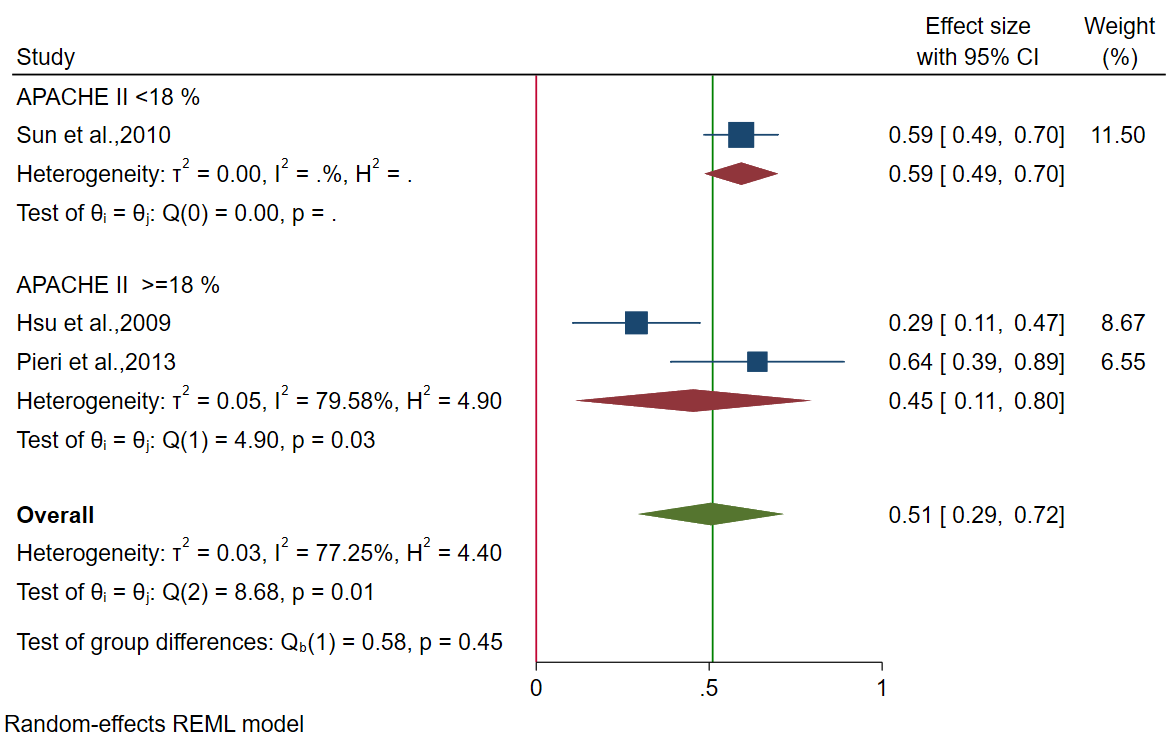 |
| --- |
| B  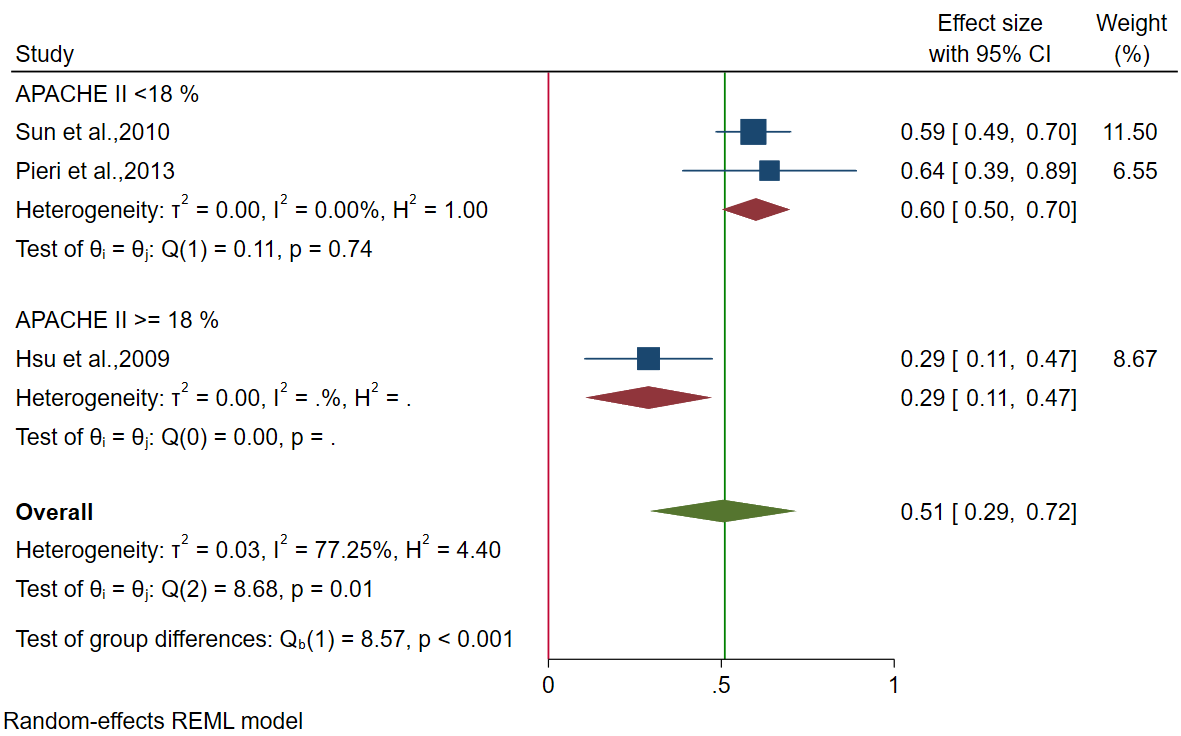 |

**Figure S1:** Forest plot of ECMO survival in all participants according to APACHEII subgroup (A) infected patients and (B) non-infected patients

| A  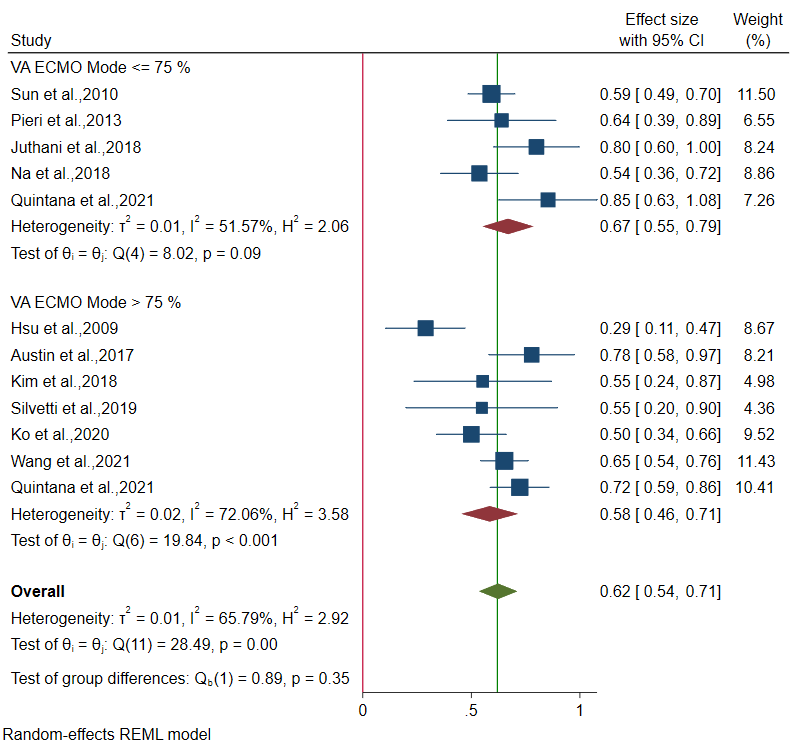 |
| --- |
| B  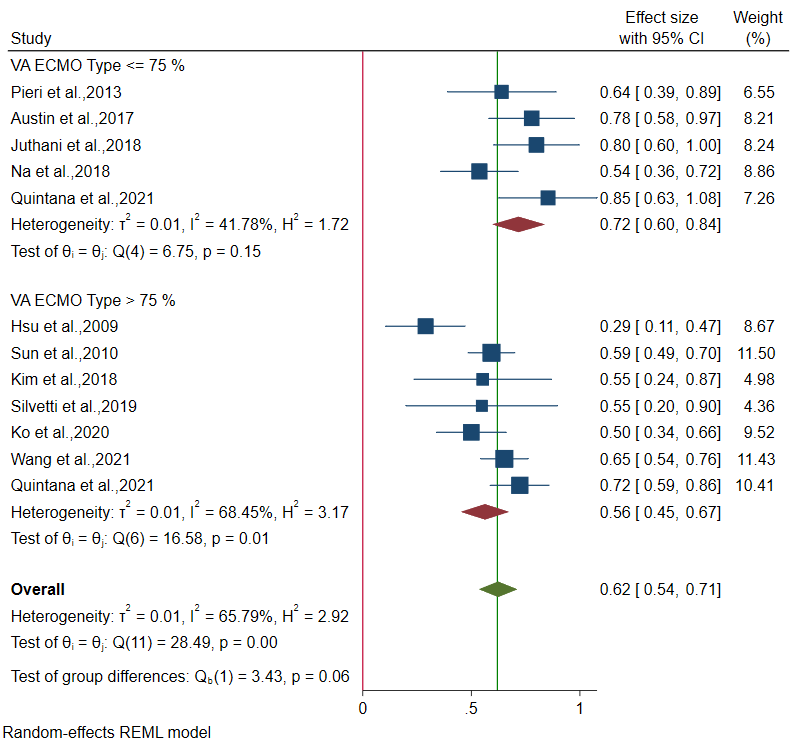 |

**Figure S2:** Forest plot of ECMO survival in all participants according to ECMO-mode subgroup (A) infected patients and (B) non-infected patients

| A  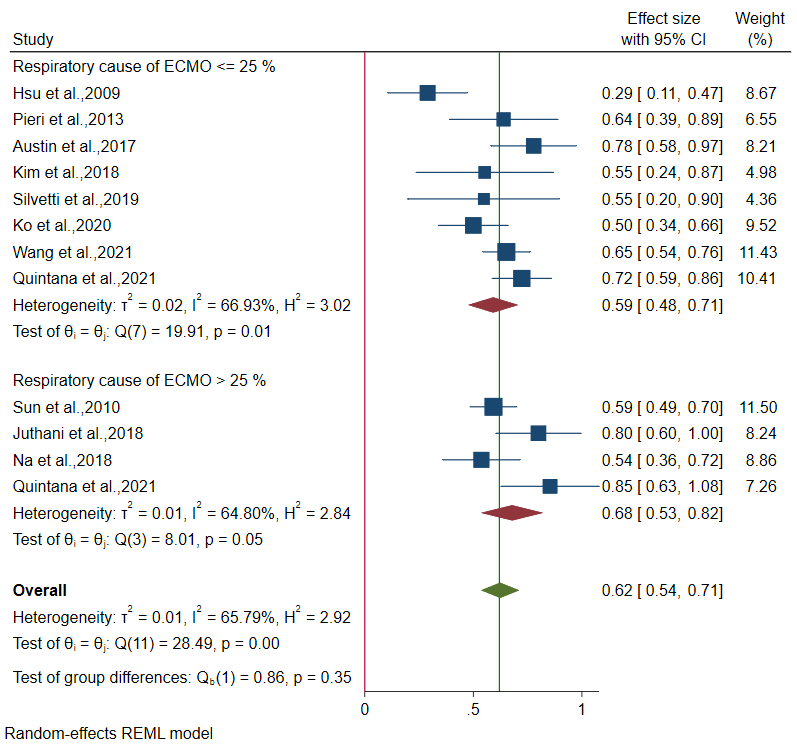 |
| --- |
| B  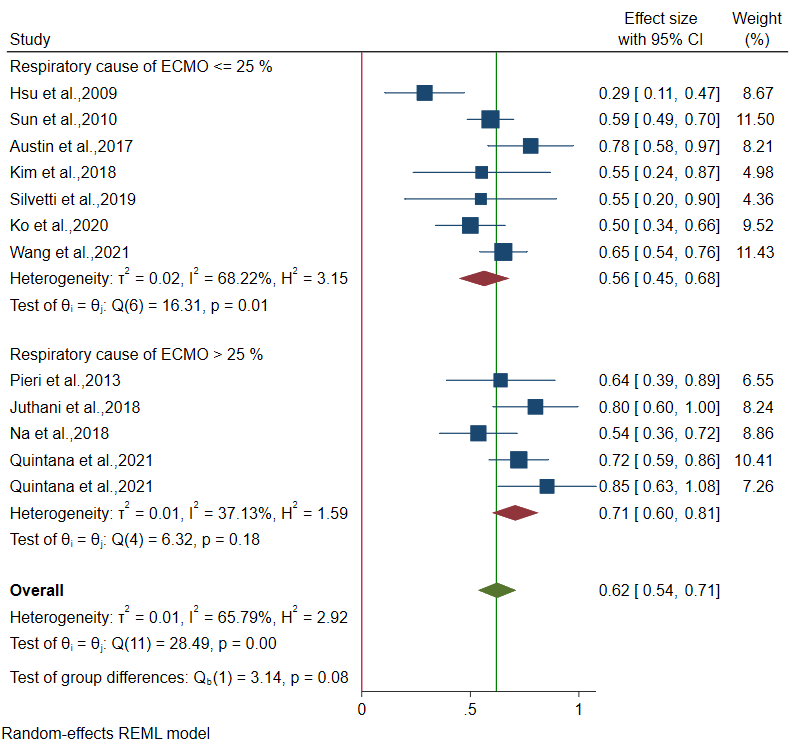 |

**Figure S3:** Forest plot of ECMO survival in all participants according to respiratory cause of ECMO subgroup (A) infected patients and (B) non-infected patients

| A  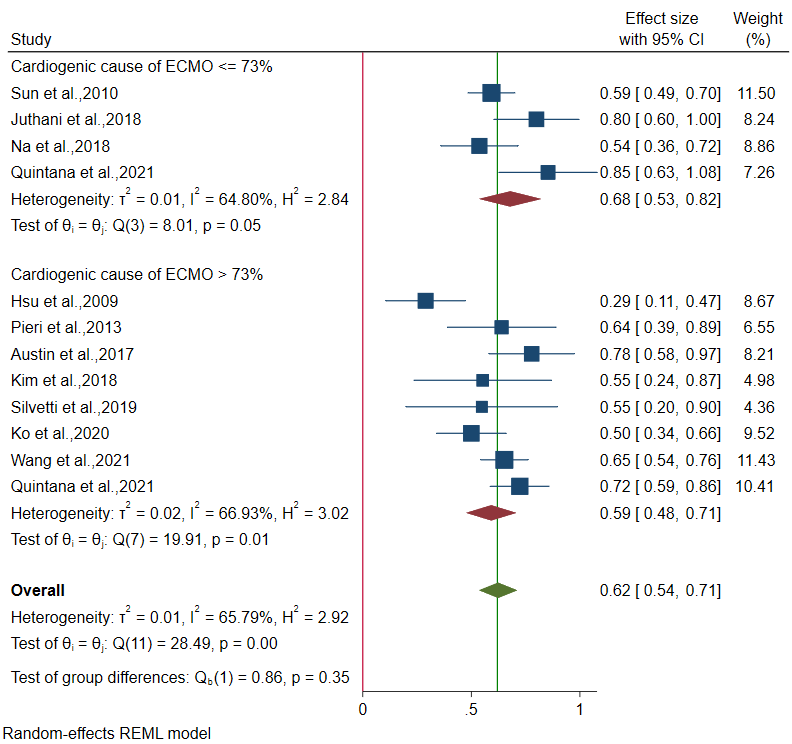 |
| --- |
| B  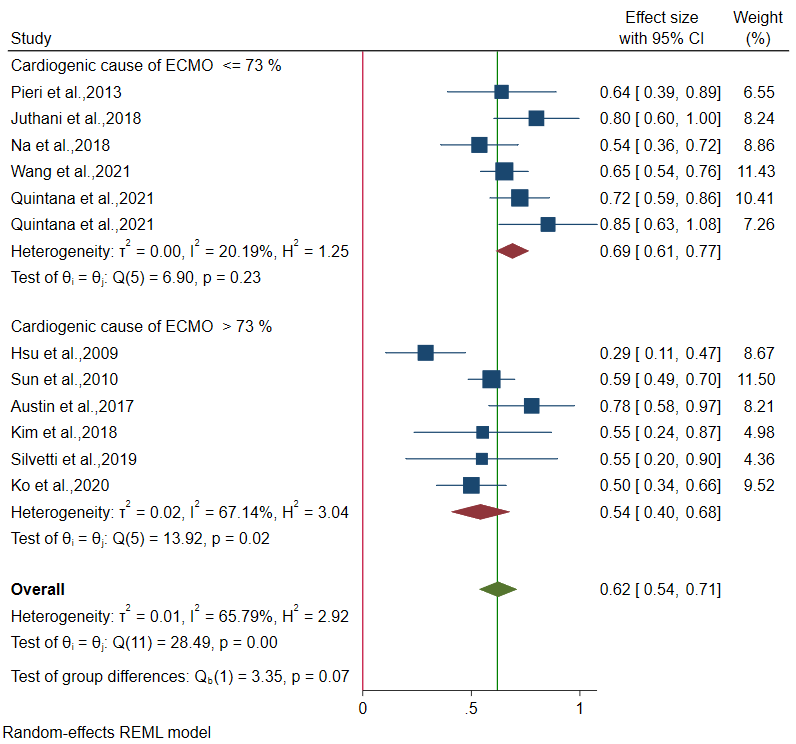 |

**Figure S4:** Forest plot of ECMO survival in all participants according to cardiogenic cause of ECMO subgroup (A) infected patients and (B) non-infected patients

| A  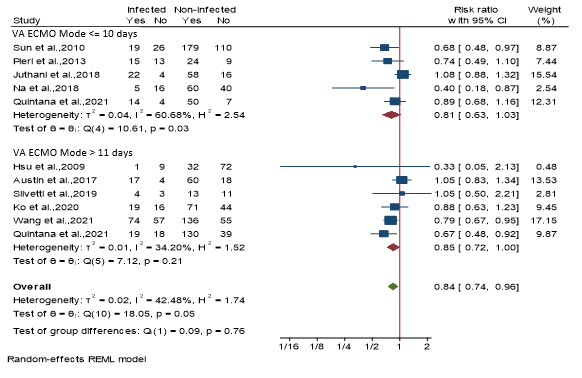 |
| --- |
| B  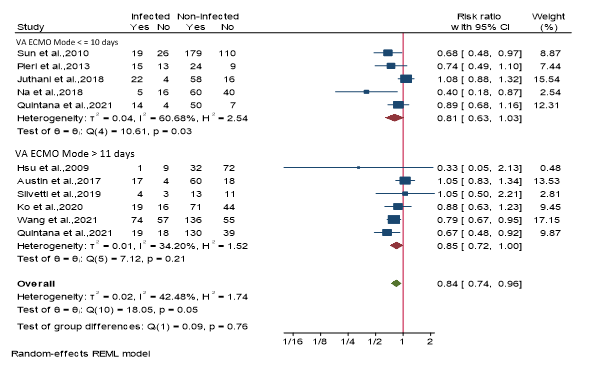 |

**Figure S5:** Forest plot of ECMO survival according to ECMO mode subgroup (A) infected patients and (B) non-infected patients


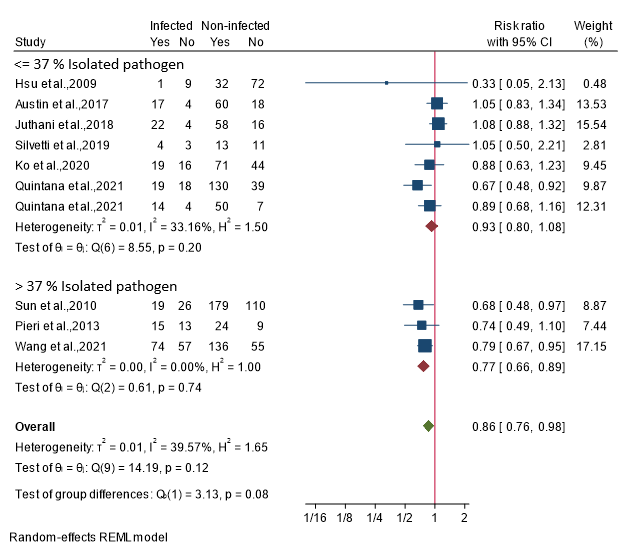


**Figure S6:** Forest plot of ECMO survival according to number of isolated pathogen subgroup in infected patients


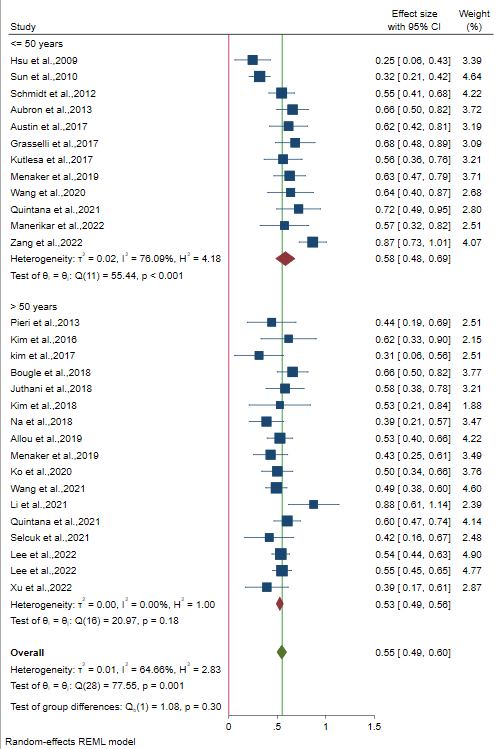


**Figure S7:** Forest plot of overall survival according to age groups (≤50 years vs. >50 years) in infected patients


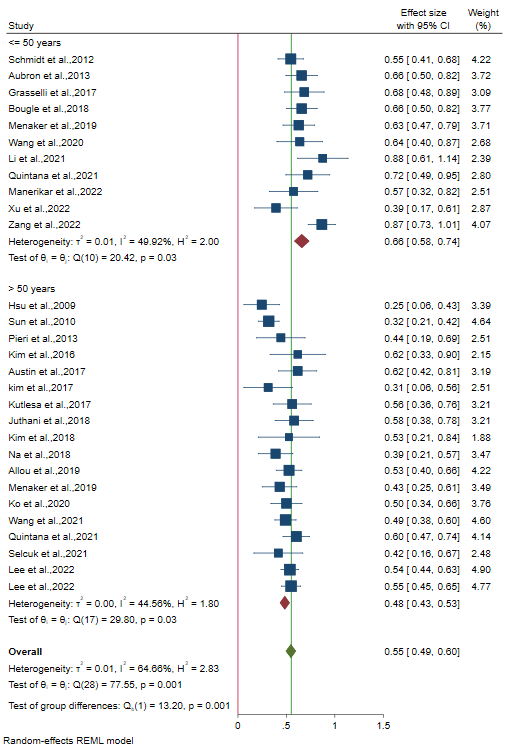


**Figure S8:** Forest plot of overall survival according to age groups (≤50 years vs. >50 years) in non-infected patients


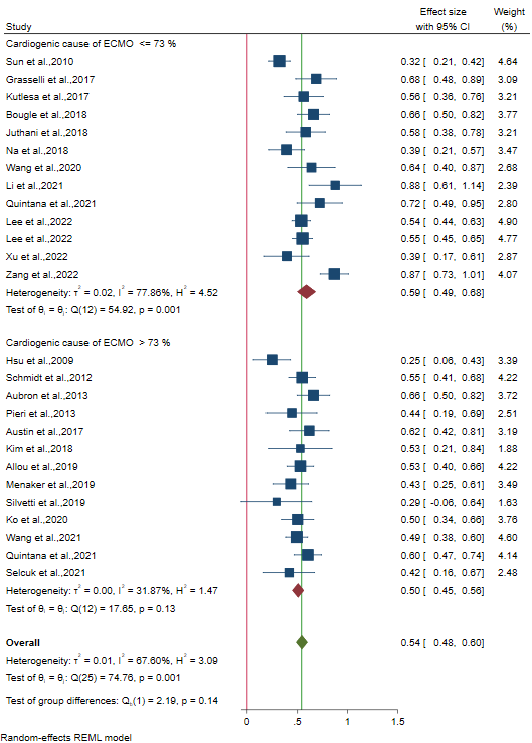


**Figure S9:** Forest plot of overall survival according to cardiogenic cause of ECMO in infected patients


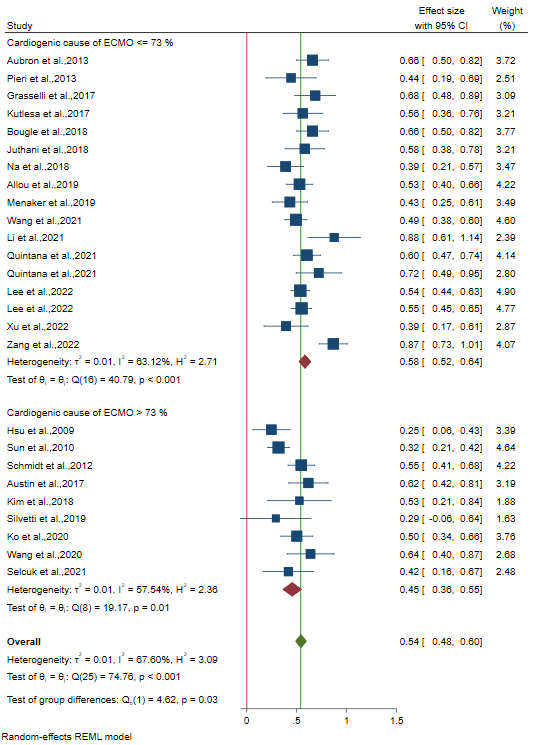


**Figure S10:** Forest plot of overall survival according to cardiogenic cause of ECMO in non-infected patients


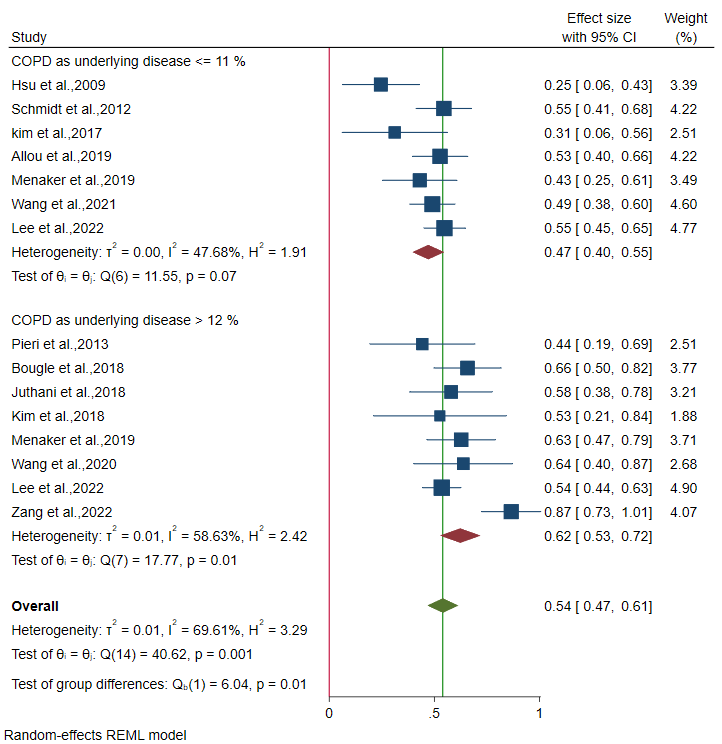


**Figure S11:** Forest plot of overall survival according to COPD (as underlying diseases) in infected patients


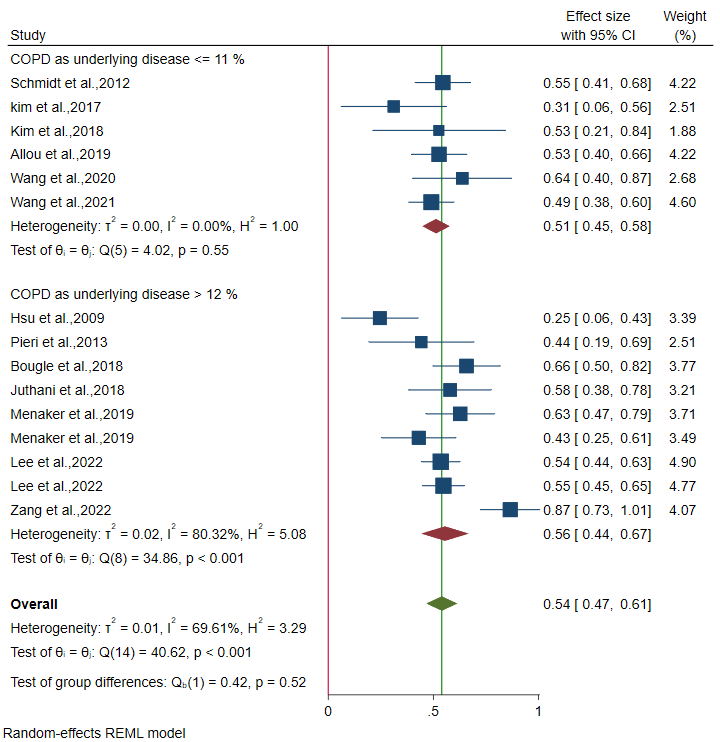


**Figure S12:** Forest plot of overall survival according to COPD (as underlying diseases) in non-infected patients


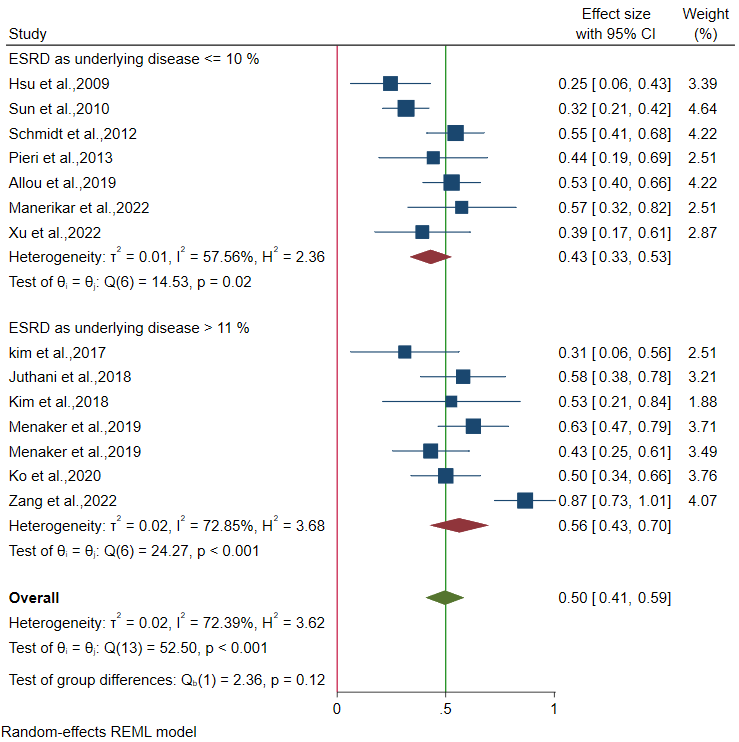


**Figure S13:** Forest plot of overall survival according to ESRD (as underlying diseases) in infected patients


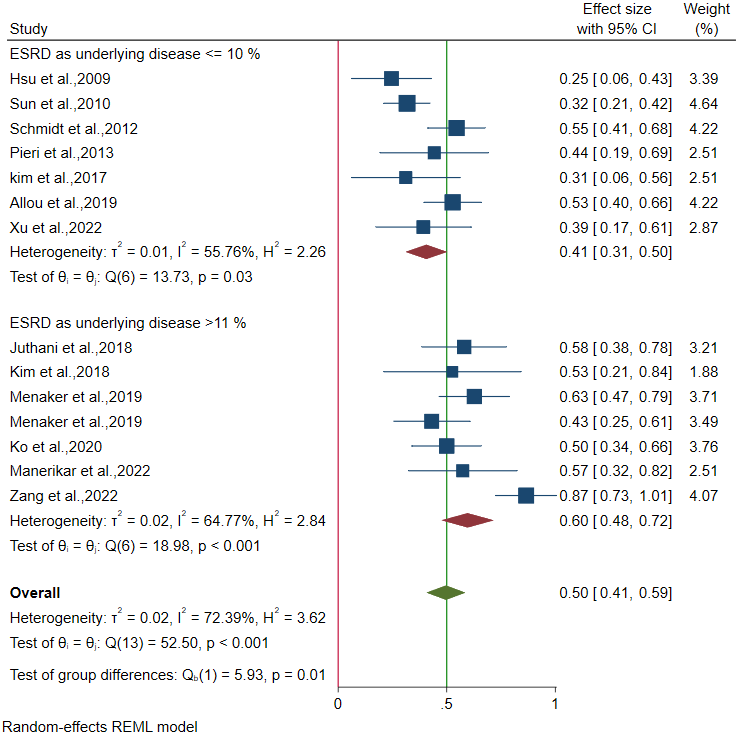


**Figure S14:** Forest plot of overall survival according to ESRD (as underlying diseases) in non-infected patients


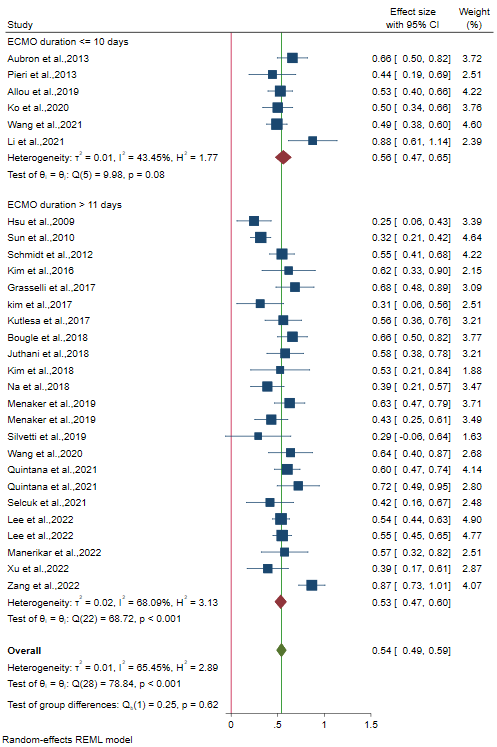


**Figure S15:** Forest plot of overall survival according to ECMO duration in infected patients


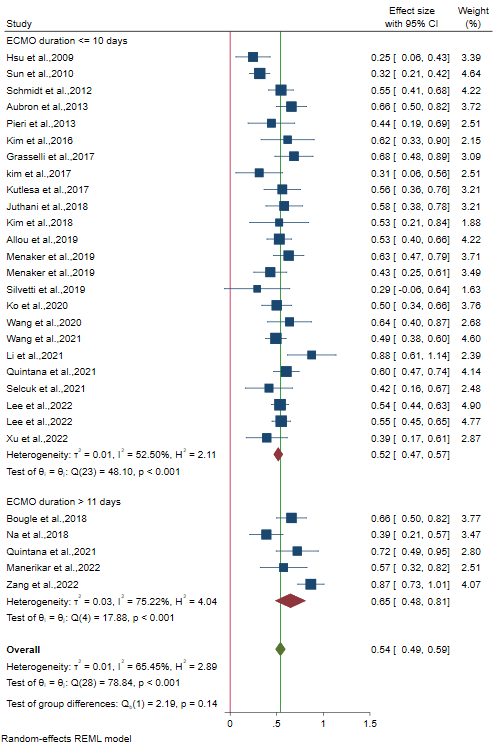


**Figure S16:** Forest plot of overall survival according to ECMO duration in non-infected patients


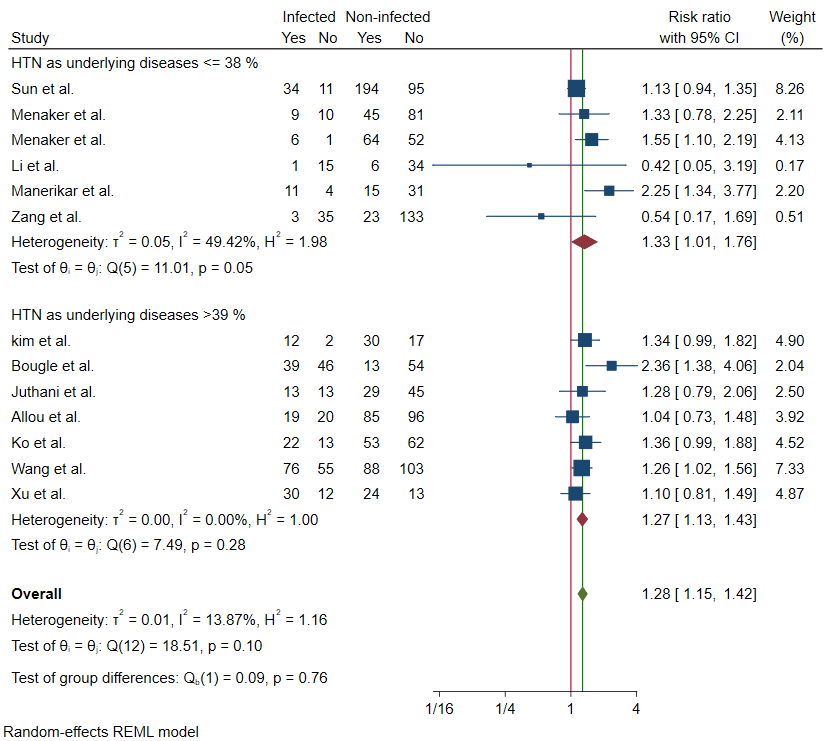


**Figure S17:** Forest plot of overall survival according to HTN (as underlying diseases) in two groups of study

**Figure S18:** Forest plot of overall survival according to ESRD (as underlying diseases) in two groups of study

**Figure S19:** Forest plot of overall survival according to respiratory cause of ECMO in two groups of study
